# LEDGF/p75-Independent HIV-1 Replication Demonstrates a Role for HRP-2 and Remains Sensitive to Inhibition by LEDGINs

**DOI:** 10.1371/journal.ppat.1002558

**Published:** 2012-03-01

**Authors:** Rik Schrijvers, Jan De Rijck, Jonas Demeulemeester, Noritaka Adachi, Sofie Vets, Keshet Ronen, Frauke Christ, Frederic D. Bushman, Zeger Debyser, Rik Gijsbers

**Affiliations:** 1 Division of Molecular Medicine, Katholieke Universiteit Leuven, Leuven, Flanders, Belgium; 2 Graduate School of Nanobioscience, Yokohama City University, Yokohama, Japan; 3 Department of Microbiology, University of Pennsylvania School of Medicine, Philadelphia, Pennsylvania, United States of America; Universitätklinikum Heidelberg, Germany

## Abstract

Lens epithelium–derived growth factor (LEDGF/p75) is a cellular cofactor of HIV-1 integrase (IN) that interacts with IN through its IN binding domain (IBD) and tethers the viral pre-integration complex to the host cell chromatin. Here we report the generation of a human somatic LEDGF/p75 knockout cell line that allows the study of spreading HIV-1 infection in the absence of LEDGF/p75. By homologous recombination the exons encoding the LEDGF/p75 IBD (exons 11 to 14) were knocked out. In the absence of LEDGF/p75 replication of laboratory HIV-1 strains was severely delayed while clinical HIV-1 isolates were replication-defective. The residual replication was predominantly mediated by the Hepatoma-derived growth factor related protein 2 (HRP-2), the only cellular protein besides LEDGF/p75 that contains an IBD. Importantly, the recently described IN-LEDGF/p75 inhibitors (LEDGINs) remained active even in the absence of LEDGF/p75 by blocking the interaction with the IBD of HRP-2. These results further support the potential of LEDGINs as allosteric integrase inhibitors.

## Author Summary

Like other viruses, HIV has a limited genome and needs to exploit the machinery of the host cell to complete its replication cycle. The elucidation of virus-host interactions not only sheds light on pathogenesis but also provides opportunities in a limited number of cases to develop novel antiviral drugs. A prototypical example is the interaction between the cellular protein LEDGF/p75 and HIV-1 integrase (IN). Here we generated a human somatic LEDGF/p75 knockout cell line to demonstrate that HIV-1 replication is highly dependent on its cofactor. We show that the residual replication of laboratory strains is predominantly mediated by a LEDGF/p75-related protein, HRP-2. Interestingly, the recently developed HIV-1 IN inhibitors that target the LEDGF/p75-IN interaction interface, LEDGINs, remain active even in the absence of LEDGF/p75. We demonstrate that LEDGINs efficiently block the interaction between IN and HRP-2. In case HIV-1 would be able to bypass LEDGF/p75-dependent replication using HRP-2 as an alternative tether, LEDGINs would remain fully active.

## Introduction

Integration of viral DNA into the host cell genome is a critical step during HIV replication. A stably inserted provirus is essential for productive infection and archives the genetic information of HIV in the host cell. The presence of a permanent viral reservoir that evades the immune system and enables HIV to rebound once antiretroviral drugs are withdrawn is one of the major remaining hurdles to surmount the HIV epidemic.

Lentiviral integration is catalyzed by the viral enzyme IN in close association with the cellular cofactor LEDGF/p75 [1]–[7]. LEDGF is encoded by the *PSIP1* gene, which generates the splice variants LEDGF/p52 and LEDGF/p75 [8]. Both share an N-terminal region of 325 residues containing an ensemble of chromatin binding elements, such as the PWWP and AT hook domain, yet differ at the C-terminus. LEDGF/p52 contains 8 amino acids at its C-terminus [9] and fails to interact with HIV-1 IN [10], [11], whereas LEDGF/p75 contains an IBD (aa 347–429) capable of interacting with lentiviral IN [3], [12], [13]. The cofactor tethers IN to the host cell chromatin, protects it from proteolytic degradation, stimulates its enzymatic activity *in vitro* and in living cells [1], [10], [13]–[16] and determines HIV-1 integration site distribution [2], [11], [17], [18].

The role of LEDGF/p75 in HIV-1 replication was studied using RNA interference (RNAi) targeting LEDGF/p75 or using LEDGF KO murine embryonic fibroblasts (MEF) [2], [5], [6], [11], [17], [19], [20]. Although both strategies point to a key role for LEDGF/p75 in lentiviral replication, they resulted in somewhat conflicting conclusions. Potent RNAi-mediated knockdown (KD) of LEDGF/p75 reduced HIV-1 replication, yet residual replication was observed [5], [6], [20], which was attributed to imperfect RNAi-mediated KD of LEDGF/p75, with minute amounts of LEDGF/p75 being sufficient to support HIV-1 replication [5], [6]. Whether LEDGF/p75 is essential for HIV-1 replication or not could not be addressed by this approach. Later, two LEDGF KO mice were generated. Since mouse cells are not permissive to spreading HIV-1 infection, HIV-based viral vectors were used. The first effort resulted in mouse LEDGF KO clones following insertion of a gene trap [21]. Data obtained from MEFs isolated from these embryos indicated a strong yet incomplete block in integration of HIV-based lentiviral vectors (LV) [17]. Next, a Cre-conditional LEDGF KO mouse was generated. Challenge of the KO MEFs with LV resulted in reduced but not annihilated reporter gene expression [11]. Although analysis was restricted to single round assays, both studies suggest LEDGF/p75 not to be essential for HIV-1 replication, with the cofactor being involved in integration site selection rather than in promoting integration. Here we present the generation of the first human somatic LEDGF/p75 KO cell line to finally answer the question whether LEDGF/p75 is required for spreading infection of various HIV strains.

Besides LEDGF/p75, a second member of the hepatoma-derived growth factor related protein family [22], Hepatoma-derived growth factor related protein 2 (HRP-2), was shown to interact with HIV-1 IN [12]. Although HRP-2 overexpression relocated IN from the cytoplasm to the nucleus in LEDGF/p75-depleted cells [23], the IN–HRP-2 interaction was weaker than the IN-LEDGF/p75 interaction [12]. Neither transient [20], [24] nor stable HRP-2 KD [6] reduced HIV-1 replication even after reduction of LEDGF/p75, suggesting that HRP-2 is not involved in HIV replication. However, it has not been excluded that in the absence of LEDGF/p75 HRP-2 can function as an alternative molecular tether of HIV integration.

Allosteric HIV-1 IN inhibitors that target the LEDGF/p75-IN interaction interface (LEDGINs) and potently block HIV-1 replication [25] are in preclinical development. The existence of alternative cellular cofactors, such as HRP-2, or alternative escape routes might hamper the clinical development of this class of compounds. To answer these questions, we have generated a human somatic LEDGF/p75 KO cell line. We demonstrate that laboratory-adapted HIV strains are capable of replicating in the absence of LEDGF/p75 but show a drastic replication defect. We show that this residual replication in the absence of LEDGF/p75 is predominantly mediated by HRP-2. Finally, we demonstrate that LEDGINs remained fully active even in the absence of LEDGF/p75 corroborating their allosteric mechanism of action.

## Results

### Generation of a human somatic LEDGF/p75 KO cell line

**Figure 1:**
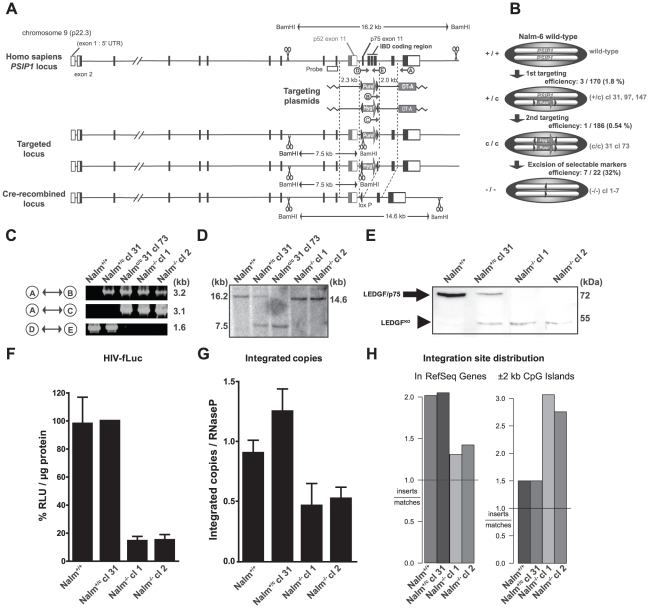
Generation and validation of human LEDGF/p75 KO cell line. (A) Scheme for *PSIP1* gene targeting by homologous recombination. The 2.3 and 2.0 kb arm indicated on the targeting plasmids enable homologous recombination and harbor a puromycin (Puro^r^) or hygromycin B resistance (Hyg^r^) cassette (c) flanked by loxP sites (arrows). Exon 11 of p52 and p75 are indicated separately as well as the IBD coding region. BamHI restriction sites are indicated (scissors). DT-A denotes the gene encoding for Diphteria toxin A. (B) Schematic overview of KO and intermediates: Nalm-6 wild-type (Nalm^+/+^) contains 2 *PSIP1* genes; Nalm^+/c^ clones (cl) 31, 97, 147, contain a puromycin resistance cassette in one allele; Nalm^c/c^ 31 cl 73, contains both a puromycin and a hygromycin B resistance cassette; after CRE-mediated excision cl 1-7 are generated and termed Nalm^−/−^. (C) Genomic PCR on DNA from different clones. Primer binding sites are indicated in panel A (see also Table S2). Indicated bands confirm amplification of a 1.6 kb fragment by primers D and E in full length *PSIP1* as shown in Nalm^+/c^ and Nalm^+/+^, but not in Nalm^c/c^ and Nalm^−/−^ cl 1. (D) Southern blot on genomic DNA after BamHI digestion. Probe and restriction sites are indicated in panel A. Intact *PSIP1* generates a 16.2 kb fragment. After insertion of a resistance cassette a 7.5 kb fragment is generated, after CRE-mediated excision a 14.6 kb fragment is formed indicating KO of a 1.6 kb fragment containing exon 11–14. (E) Western blot analysis for LEDGF protein of whole cell extracts. Marker heights (right), LEDGF/p75 (arrow) and LEDGF^KO^ (arrowhead) are indicated. (F) Nalm-6 cells were transduced with HIV-fLuc. Luciferase expression is shown as percentage relative light units (RLU) per µg protein as compared to Nalm^+/c^ cl 31. (G) In parallel, the number of integrated proviral copies was evaluated for HIV-fLuc. Following transduction, cells were grown for at least 10 days to eliminate non-integrated viral DNA and analyzed by quantitative PCR. (H) HIV-1 integration site distribution analysis. Left panel shows relative number of experimentally derived HIV-1 integration events in genes according to the RefSeq annotation, versus computationally generated matched random control (MRC). The right panel shows integration events occurring ±2 kb around CpG islands as compared with MRC. Average ± standard deviations are shown from experiments performed at least in triplicate.

To clarify the role of LEDGF/p75 during spreading HIV-1 infection, we generated a human somatic KO in Nalm-6 cells, a human pre-B acute lymphoblastic leukemia cell line [26], [27]. We eliminated the LEDGF/p75 isoform while preserving the LEDGF/p52 splice variant. Deletion of exon 11 to 14 in the *PSIP1* gene fuses exon 10 to exon 15 resulting in a frame shift that yields a truncated LEDGF/p75 in which the C-terminus, including the IBD (aa 326–530) is replaced by a 9 aa tail (Figure S1A, referred to as LEDGF^KO^). Targeting plasmids were designed carrying the genomic flanking regions of LEDGF/p75 exon 11 and 14, interspersed with a floxed selection cassette (Figure 1A). Following transfection of wild-type Nalm-6 cells (Nalm^+/+^) with the first targeting plasmid and subsequent selection, three heterozygous clones (cl) (denoted as Nalm^+/c^; cl 31, cl 97 and cl 147, respectively) were obtained (Figure 1B). We continued with Nalm^+/c^ cl 31. Transfection of Nalm^+/c^ cl 31 with the second targeting plasmid resulted in the selection of a homozygous KO clone carrying both resistance cassettes (Nalm^c/c^ 31 cl 73). Selection cassettes were removed by Cre-mediated excision, resulting in seven LEDGF/p75 KO clones, referred to as Nalm^−/−^ cl 1-7.

Correct homologous recombination of the genomic region was verified via genomic PCR (Figure 1C), Southern blot analysis (Figure 1D) and sequencing of the genomic and mRNA region (Figure S1A). The absence of wild-type LEDGF/p75 in the KO cells was corroborated by RT-PCR (Figure S1B and S1C), qRT-PCR (Figure S1D) and Western blot analysis (Figure 1E, arrow). A band of 52 kDa appears in the Nalm^+/c^ and Nalm^−/−^ cell lines; it corresponds to the truncated form, LEDGF^KO^ (Figure 1E, arrowhead), and is absent in wild-type cells. Throughout the manuscript Nalm^−/−^ cl 1 and cl 2 monoclonal cell lines are used. Wild-type Nalm-6 cells, referred to as Nalm^+/+^, were used as controls, next to Nalm^+/c^ cl 31, referred to as Nalm^+/c^, the closest clonal ancestor of the Nalm^−/−^ cells.

### Single round lentiviral transduction of LEDGF/p75 KO cells is hampered at the integration step

We first evaluated whether the LEDGF/p75 KO cells (Nalm^−/−^) support transduction by a single round HIV-based viral vector. We challenged the abovementioned engineered cell lines with a VSV-G pseudotyped HIV reporter virus encoding firefly luciferase under control of the viral long terminal repeat promoter (HIV-fLuc). Transduction efficiency (RLU/µg protein) was 6.7-fold lower in Nalm^−/−^ cells (cl 1 and cl 2) compared to control Nalm^+/+^ and Nalm^+/c^ cells (Figure 1F) (15±3.7% residual reporter activity; n = 10). Quantitative PCR revealed 2.4-fold lower integrated copies comparing Nalm^−/−^ with Nalm^+/c^ (Figure 1G), whereas late RT products (Figure S1E) and 2-LTR circles remained unaffected (Figure S1F). Together these data indicate a block between reverse transcription and integration.

Since LEDGF/p75 determines lentiviral integration site selection, we analyzed the distribution of HIV-1 integration sites in the absence of LEDGF/p75. A total of 2535 HIV-1 integration sites were obtained in Nalm-6 cells of which 799 in Nalm^−/−^ (Table 1). Random control sites were generated computationally and matched to experimental sites with respect to the distance to the nearest *MseI* cleavage site (matched random control, MRC) [2]. LEDGF/p75 KO significantly reduced the preference of HIV-1 to integrate in RefSeq genes (*P*<0.0001 for comparison of Nalm^−/−^ cl 1 or 2 with Nalm^+/+^ or Nalm^+/c^) and instead, a preference for CpG islands (*P*<0.05 for comparison of Nalm^−/−^ cl 1 or 2 with Nalm^+/+^ or Nalm^+/c^ and *P*<0.0001 for pooled comparison) emerged (Figure 1H and Table 1). Similar results were obtained using the Ensembl and UniGene annotation (Figure S1G and S1H). HIV-1 integration events in RefSeq genes remained nevertheless significantly favored over MRC in the KO cells (*P*<0.0001). The target DNA consensus proved to be LEDGF/p75 independent (compare Figure S1I with S1J). The consensus sequence for the different cell lines was similar to that determined previously [28]–[30].

**Table 1:** Integration frequency near mapped genomic features in the human genome.

	Cell line	# sites	% in RefSeq genes	%±2 kb CpG islands
**HIV sites**	Nalm^+/+^	1075	76.7**	4.2
	Nalm^+/c^	661	78.8**	5.0
	Nalm^−/−^ cl 1	404	52.2**	11.1**
	Nalm^−/−^ cl 2	395	51.4**	8.6**
**MRC sites**	Nalm^+/+^	3225	39.8	2.8
**(HIV)**	Nalm^+/c^	1983	39.8	3.3
	Nalm^−/−^ cl 1	1212	40.8	3.6
	Nalm^−/−^ cl 2	1185	37.4	3.1

*Abbreviations:* MRC, matched random control.

Significant deviation from MRC using a two-tailed Fisher's exact test (with Bonferroni correction) is denoted by *P<0.00625, **P<0.0001.

### In LEDGF/p75 KO cells residual replication is observed with laboratory strains but not with clinical isolates of HIV-1

**Figure 2:**
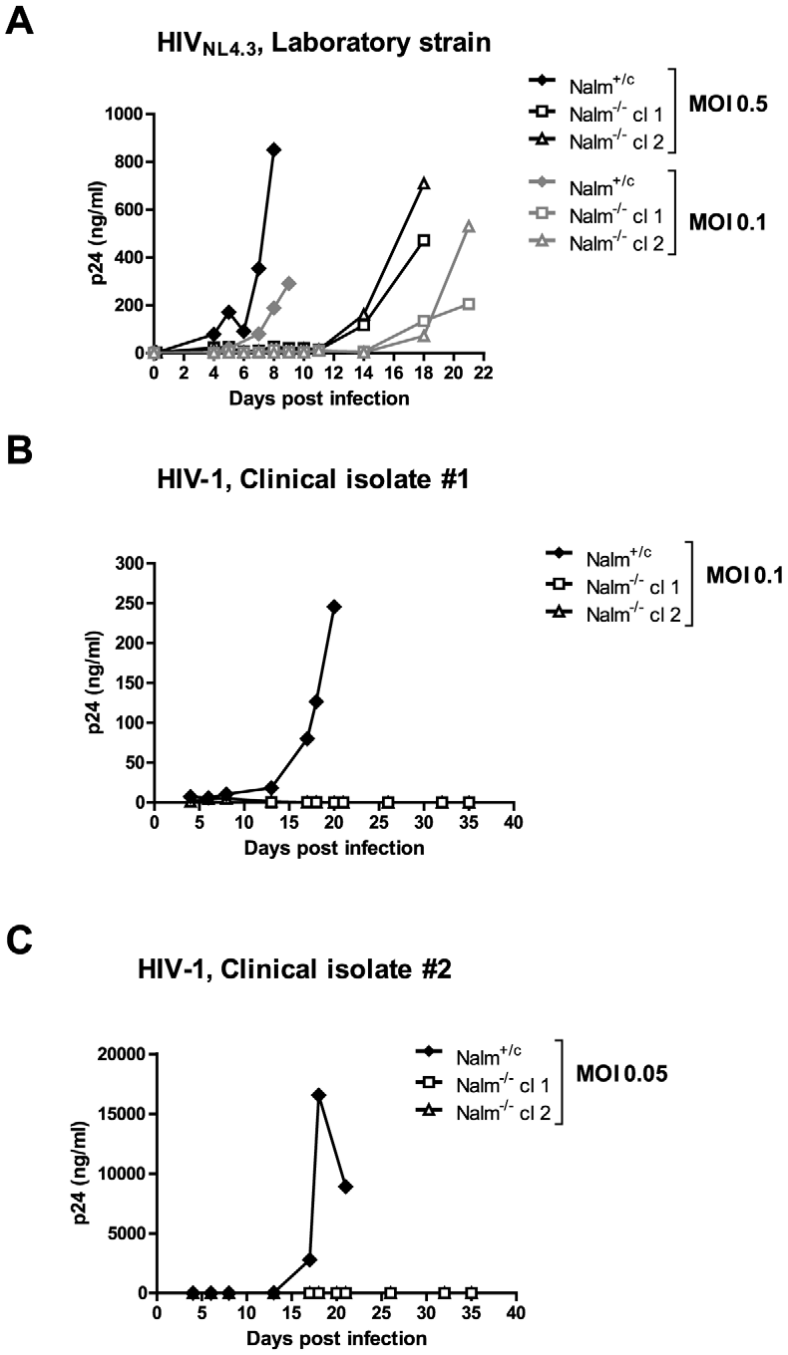
Residual HIV-1 replication in LEDGF/p75 KO cells is only observed after challenge with laboratory strains. Control Nalm^+/c^ and KO Nalm^−/−^ cl 1 and cl 2 cell lines were challenged with the laboratory strain HIV_NL4.3_ (A) and clinical isolates #1, 93TH053 (B) and #2, 96USSN20 [32] (C). Cells were infected at different MOI as indicated. Replication was monitored by measuring the p24 content of the supernatant. Experiments were repeated at least three times; representative experiments are shown.

In human LEDGF/p75 KD cells HIV-1 replication is hampered, but not completely blocked which can be attributed to the remaining minute amounts of LEDGF/p75 [5], [6], [20]. Although single round viral vector transduction was severely reduced in LEDGF KO MEFs [11], [17], [21], spreading HIV-1 infection in the absence of LEDGF/p75 could not be tested. To test HIV-1 replication, we introduced the CD4 receptor into the Nalm-6 cells that express CXCR4 [31], a co-receptor for HIV-1 replication. All selected transgenic cell lines (Nalm^+/+^, Nalm^+/c^ and Nalm^−/−^ cl 1 and cl 2) showed similar growth rates (Figure S6A and S6C) and CD4 and CXCR4 expression levels (Figure S6D and S6E). We then challenged the respective cell lines with the laboratory strain HIV_NL4.3_ (Figure 2A). Both Nalm^+/+^ (Figure S2A) and Nalm^+/c^ cells supported viral replication to the same extent (Figure 2A). Peak viral replication was consistently observed between day 7 and 9 post infection depending on the multiplicity of infection (MOI; compare MOI 0.5 and 0.1 in Figure 2A). In Nalm^−/−^ cells infected with HIV_NL4.3_, low-level p24 production was observed, eventually leading to a breakthrough albeit after a lag-period of 14 to 18 days compared to control cells (Figure 2A, n = 6, a representative experiment is shown). Comparable data showing this delay were obtained with another laboratory strain, HIV_IIIb_ (data not shown).

Next, we challenged the different cell lines with two clinical isolates of HIV-1 (93TH053, denoted as #1 and 96USSN20 [32], denoted as #2). Viral breakthrough was observed 17 to 20 days post infection in the control cell line (Figure 2B and 2C). In the first two weeks after infection of the KO cell lines only a discrete increase in p24 was observed; at 35 days after infection p24 levels were below detection limit (Figure 2B and 2C).

### Residual virus production in KO cells is caused by spreading infection which is hampered at the integration step

We next evaluated whether the rise in p24 titers observed in Nalm^−/−^ cells after challenge with laboratory HIV-1 strains could be explained by virus release from cells infected in the first round, rather than ongoing replication cycles. Therefore we challenged Nalm^+/c^ and Nalm^−/−^ cells with HIV_NL4.3_ and resuspended the cells at 8 hrs post infection (Figure S2B) in fresh medium containing either zidovudine (AZT), ritonavir (RIT) or no inhibitor. AZT, a reverse transcriptase inhibitor, blocks infection of new cells but allows monitoring of virus release from already infected cells whereas RIT, a protease inhibitor, blocks processing of GAG-precursor processing in the virus released from infected cells. In Nalm^−/−^ cells as well as in control Nalm^+/c^ cells the p24 production clearly decreased in the presence RIT or AZT. The decrease in p24 in Nalm^+/c^ without inhibitor at day 6 was due to the cytopathic effect of the virus. This indicates that the p24 increase observed in Nalm^−/−^ cells results from spreading infection and not solely from virus release from cells infected in the first round.

The observed delay in multiple round HIV-1 replication in the absence of LEDGF/p75 was further analyzed by quantification of the different HIV-1 DNA species at different time points after infection. Late RT products at 10 hrs post infection and 2-LTR circles at 24 hrs post infection were comparable in Nalm^+/c^ and Nalm^−/−^ cells (Figure S3A and S3B). Addition of the IN strand transfer inhibitor (INSTI) raltegravir (RAL) in Nalm^+/c^ and Nalm^−/−^ cell lines resulted in a comparable increase in 2-LTR circles at 24 hrs post infection. The number of integrated proviral copies (*Alu*-qPCR, Figure S3C) was severely reduced in the presence of RAL. In Nalm^−/−^ a reduction in the number of integrants was detected after 24 and 48 hrs compared to Nalm^+/c^ cell lines.

We next characterized the virus harvested from Nalm^−/−^ at day 18 after infection with the laboratory strain HIV_NL4.3_ (referred to as HIV^−/−^). Challenging Nalm^+/c^ cells with this virus demonstrated that HIV^−/−^ is replication competent (Figure S2C, right panel, HIV^−/−^ on Nalm^+/c^). In addition, we evaluated whether HIV^−/−^ virus was phenotypically adapted to the absence of LEDGF/p75. HIV^−/−^ replication remained impaired in Nalm^−/−^ compared to Nalm^+/c^ cells (Figure S2C, right panel). The proviral IN sequence of HIV^−/−^ was unaltered compared with the consensus sequence of HIV_NL4.3_ (data not shown). Control HIV harvested from Nalm^+/c^ cells (denoted as HIV^+/c^) demonstrated a phenotype that was comparable to that of HIV_NL4.3_ (Figure S2C, left panel). Serial passaging (N = 10) of HIV-1 on LEDGF/p75 KO cells did not result in phenotypic adaptation or changes in the proviral IN sequence (data not shown).

### HRP-2 KD inhibits residual HIV-1 replication in LEDGF/p75 KO cells

**Figure 3:**
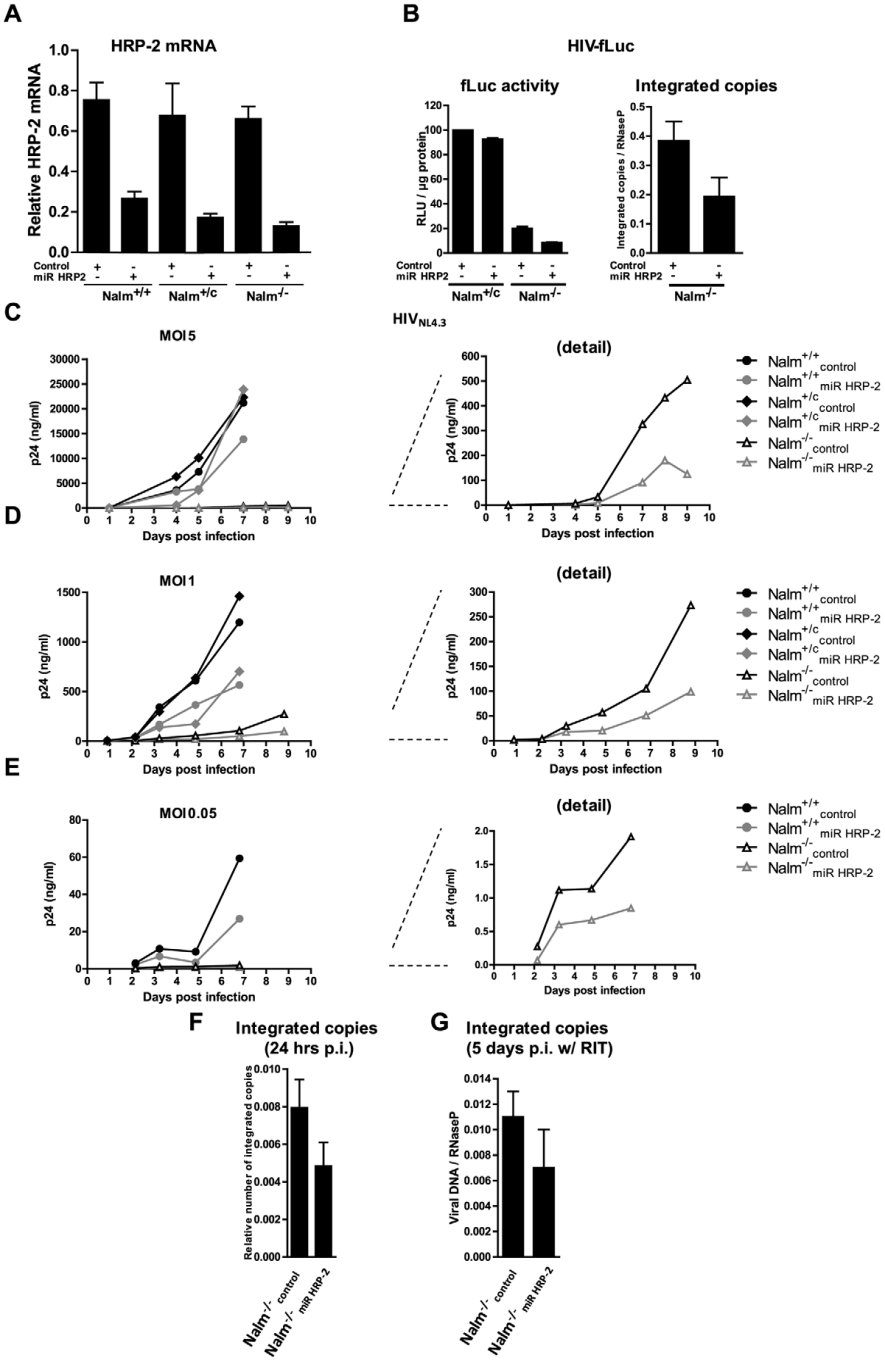
Additional HRP-2 KD hampers residual replication in LEDGF/p75 KO cells. Stable HRP-2 KD (miR HRP-2) and control (control) Nalm^+/+^, Nalm^+/c^ and Nalm^−/−^ were generated. (A) HRP-2 mRNA levels were determined by qPCR. (B) In the left panel luciferase activity following HIV-fLuc transduction is shown. Cells were grown for an additional 10 days to eliminate all non-integrated viral DNA species before total viral DNA was measured as demonstrated in the right panel. (C,D,E) Stable cell lines were challenged with the laboratory strain HIV_NL4.3_ at different MOIs (5-0.05). Replication was monitored by measuring the p24 content of the supernatant. In (F) the number of integrated copies as determined by *Alu*-qPCR is shown. In (G) we quantified total viral DNA at 5 days post infection, in the presence of Ritonavir (RIT), added 4 hrs post infection. Average ± standard deviations are shown from experiments performed at least in triplicate.

Although residual HIV-1 replication in KO cells was only detectable after infection with laboratory strains, we performed additional experiments to understand this phenotype. Residual viral replication in the absence of LEDGF/p75 can either be explained by cofactor independent replication, or by the presence of a second cofactor that substitutes for LEDGF/p75. Like LEDGF/p75, HRP-2 also harbors a PWWP-domain and an IBD-like domain shown to interact with HIV-1 IN *in vitro* [12]. In order to determine whether HRP-2 can act as an alternative co-factor for HIV integration, we targeted the HRP-2 mRNA using miRNA-based short hairpins (miR HRP-2). As controls we employed a vector lacking the miRNA expression cassette (denoted as control) (Figure S7B). We generated stable HRP-2 KD cells, termed Nalm^+/+^_miR HRP-2_, Nalm^+/c^_miR HRP-2_ and Nalm^−/−^_miR HRP-2_ and matched controls Nalm^+/+^_control_, Nalm^+/c^_control_ and Nalm^−/−^_control_. HRP-2 KD cells showed 65, 75 and 80% depletion of HRP-2, respectively, as determined by qPCR (Figure 3A). No effect on cellular growth kinetics was observed (data not shown). Upon single round transduction with HIV-fLuc no difference was observed in Nalm^+/c^ cells with or without HRP-2 KD (Figure 3B, left panel), whereas luciferase activity was reduced 5-fold in the Nalm^−/−^_control_ cell line (20.0±1.5%, n = 3) due to LEDGF/p75 KO. An additional 2.4-fold reduction was observed in Nalm^−/−^_miR HRP-2_ when compared to Nalm^−/−^_control_ (8.4±0.6%, n = 3) (Figure 3B, left panel) that correlated with a 2-fold reduction in integrated copies (Figure 3B, right panel).

We next challenged these cells with the laboratory strain HIV_NL4.3_ at different MOI (Figure 3C–E). In the control Nalm^+/+^ and Nalm^+/c^ cell lines, we observed a minor reduction in viral replication upon HRP-2 KD but only at lower MOI (compare Figure 3C and 3D, E). However, HRP-2 KD in LEDGF/p75 KO cells additionally inhibited HIV-1 replication 2- to 3-fold compared to control cells (Figure 3C–E, compare Nalm^−/−^_control_ and Nalm^−/−^_miR HRP-2_, detail panel). We generated a second LEDGF/p75 KO HRP-2 KD cell line to corroborate our results. Single round transduction with HIV-fLuc resulted in an additional 4.7-fold reduction of luciferase reporter activity when compared with LEDGF KO cells (Figure S5F), whereas HIV_NL4.3_ replication was affected 10-fold at day 8 post infection when comparing LEDGF/p75 KO and LEDGF/p75 KO HRP-2 KD cells (compare Figure S5D with S5E, condition without compounds). To corroborate that additional KD of HRP-2 results in an increased block of integration in LEDGF/p75 KO cells, we analyzed the number of integrated viral copies at 24 hrs and at 5 days post infection, the latter in the presence of RIT (Figure 3F and 3G, respectively). A 2-fold drop in proviral copies upon HRP-2 KD was observed.

### HRP-2 KD further hampers HIV-1 replication in LEDGF/p75 KD cells

**Figure 4:**
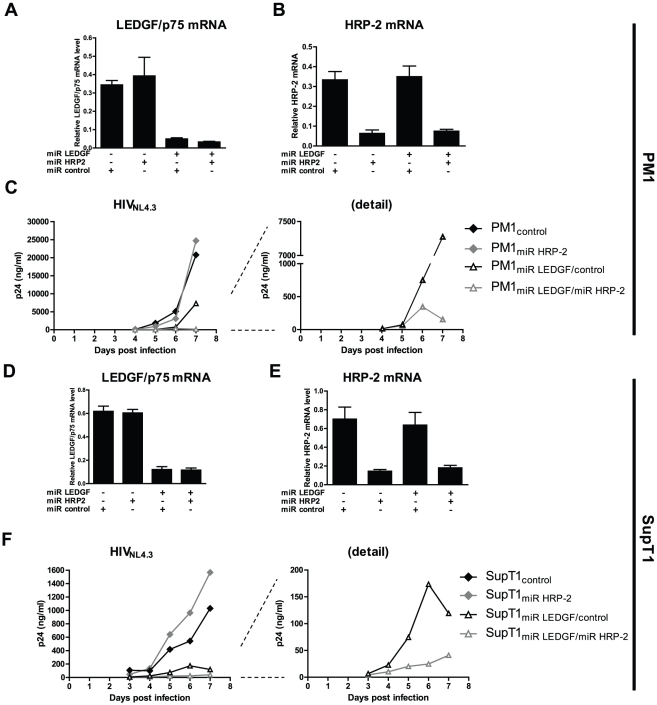
HRP-2 KD additionally hampers HIV-1 replication in LEDGF/p75 KD cell lines. Stable LEDGF/p75 and/or HRP-2 KD and control PM1 and SupT1 cells were generated. In (A) qPCR results for LEDGF/p75 and (B) HRP-2 mRNA expression levels, normalized to RNaseP for PM1 are shown. Average with standard deviations from experiments in triplicate, are shown. The constructs used, are shown below the graph. (C) Multiple round HIV-1 replication after challenge with the laboratory strain HIV_NL4.3_. On the right a detail panel for LEDGF/p75 KD cells is shown. In (D) LEDGF/p75 and (E) HRP-2 expression levels in SupT1 cells, analogous to (A) and (B) are shown. (F) Multiple round HIV-1 replication in the different SupT1 cells, analogous to (C), is shown.

To extend our findings in LEDGF/p75 KO cells, we tested whether HRP-2 KD resulted in additional reduction of viral replication in LEDGF/p75 KD HeLaP4 (Figure S4), PM1 (Figure 4A–C) and SupT1 (Figure 4D–F) cell lines. First, wild-type HeLaP4 (wild-type) and LEDGF/p75 KD (miR LEDGF) cells [18] were transduced with miR HRP-2 or miR control vectors, the latter containing a miRNA-hairpin directed against monomeric red fluorescent protein (DsRed) mRNA [33] (Figure S7C). Following zeocin selection, single HRP-2 KD (wild-type/miR HRP-2) and double KD (miR LEDGF/miR HRP-2) cells showed 20–25% of residual HRP-2 mRNA levels compared to the control cell lines (wild-type, wild-type/miR control and miR LEDGF/miR control cells) as determined by qPCR (Figure S4A and S4C). Loss of HRP-2 protein was corroborated by Western blot analysis and immunocytochemistry (data not shown). Of note, LEDGF/p75 levels remained unaffected upon additional HRP-2 KD (data not shown) and growth rates of the respective cell lines were comparable (Figure S6B and S6C). KD of HRP-2 in wild-type HeLaP4 cells did not affect multiple round HIV-1 replication (Figure S4B), confirming previous findings by Llano et al. [6]. LEDGF/p75 KD on the other hand reduced HIV-fLuc transduction 5-fold (luciferase reporter activity = 19.2±3.5% of wild-type) (Figure S4D). Additional KD of HRP-2 in LEDGF/p75-depleted cells diminished HIV-fLuc reporter activity an additional 3-fold, to 6.3±2% of control cells (miR LEDGF/miR control) (Figure S4D). This reduction was accompanied with a 2-fold decrease in the number of integrated copies (Figure S4E). Transfection of the cell lines with the plasmid encoding HIV-fLuc (pHIV-fLuc) did not demonstrate any difference (Figure S4F), ruling out transcriptional effects upon HRP-2 KD. Next, we infected double KD (miR LEDGF/miR HRP-2) cells and control (miR LEDGF/miR control) cells together with wild-type and LEDGF/p75 back-complemented (LEDGF BC) cells with the laboratory strain HIV_NL4.3_ (Figure S4G). Viral replication was inhibited in miR LEDGF cells and rescued upon LEDGF/p75 back-complementation (Figure S4G, compare wild-type and LEDGF BC). Additional KD of HRP-2 in LEDGF/p75 depleted cells (miR LEDGF/miR HRP-2) inhibited viral replication more than LEDGF/p75 KD alone (miR LEDGF/miR control). The latter demonstrated a breakthrough around day 30 post infection (Figure S4G, open diamonds), whereas cells with double KD did not demonstrate viral breakthrough (Figure S4G, open squares). Analysis was ended at 48 days post infection. Comparable data were obtained in HeLaP4 cell lines generated with other LV constructs (Figure S7B and S7D) using hygromycin B selection or eGFP sorting (data not shown). The additional block of HIV-1 replication upon HRP-2 KD in LEDGF/p75 depleted cell lines was also measured by quantifying the number of integrated proviral copies. At day 39, 45 and 48 post infection the number of integrated copies was low in double KD (miR LEDGF/miR HRP-2) cells compared to the control LEDGF/p75 KD (miR LEDGF/miR control) cells (Figure S4H) with proviruses numbering 0.032 (±0.012) and 0.038 (±0.012) per RNaseP genomic copy on day 39 and 48 respectively, compared to 1.39 (±0.18) and 0.79 (±0.23) in the control LEDGF/p75 KD cell lines. In addition, we quantified different HIV-1 DNA species at different time points post infection in wild-type, LEDGF/p75 KD (miR LEDGF/miR control) and double KD cells (miR LEDGF/miR HRP-2). We observed no difference in late RT products at 10 hrs post infection (Figure S4I). The number of 2-LTR circles in LEDGF/p75 KD (miR LEDGF/miR control) and both LEDGF/p75 and HRP-2 KD (miR LEDGF/miR HRP-2) cells was elevated compared to wild-type cells (Figure S4J). Together with the data in the LEDGF/p75 KO cells, these data indicate that HRP-2 KD blocks HIV-1 at a step between reverse transcription and integration but only after potent depletion of LEDGF/p75.

Next, we expanded our findings to relevant T-cell lines, PM1 and SupT1. We generated cell lines with stable KD of LEDGF/p75, HRP-2 or both, together with their respective controls (constructs shown in Figure S7B). For PM1 cells KD efficiency was 85–92% for LEDGF/p75 (Figure 4A) and 79–81% for HRP-2 (Figure 4B), for SupT1 cells it amounted to 81–88% for LEDGF/p75 (Figure 4D) and 75–80% for HRP-2 (Figure 4E). In both cell lines HRP-2 KD alone did not affect HIV-1 replication, whereas a clear reduction in HIV-1 replication was observed upon LEDGF/p75 KD (Figure 4C and 4F, left panel, for PM1 and SupT1 respectively). Consistent with our findings in LEDGF/p75 KO cells and LEDGF/p75 depleted HeLaP4 cells, also in PM1 and SupT1 cells, HRP-2 KD in LEDGF/p75 depleted cells further hampered HIV-1 replication (Figure 4C and 4F, detail panels, for PM1 and SupT1 respectively).

### LEDGINs block residual HIV-1 replication in Nalm^−/−^ cells

**Figure 5:**
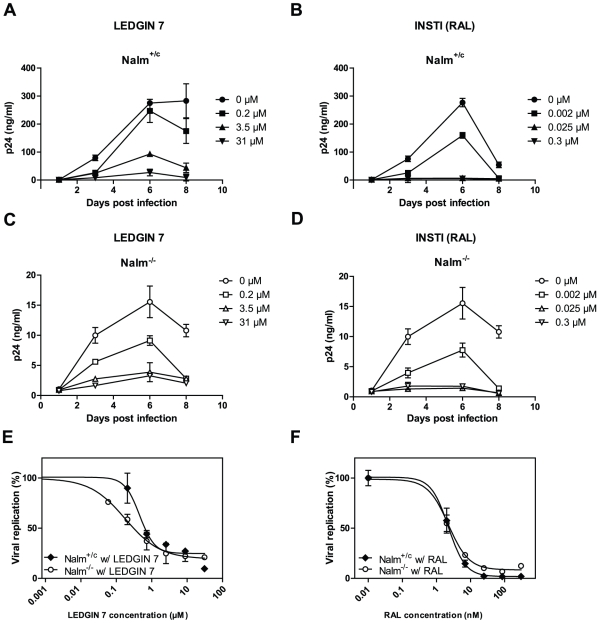
LEDGINs block residual HIV-1 replication in LEDGF/p75 KO cells. Nalm^+/c^ and Nalm^−/−^ cell lines were challenged with HIV_IIIb_ in the presence of varying concentrations of HIV inhibitors. Supernatant was harvested for p24 ELISA. (A,C) LEDGIN 7 was added at different concentrations (0 µM, circles; 0.2 µM, squares; 3.5 µM, triangles or 31 µM, triangles pointing downwards). (B,D) Identical conditions were used as shown in (A,C), but the INSTI raltegravir (RAL) was added at different concentrations (0 µM, circles; 0.002 µM, squares; 0.025 µM, triangles or 0.3 µM, triangles pointing downwards). (A–D) Averages from duplicate experiments ± standard deviations are shown. (E) Dose-response curves in Nalm^+/c^ and Nalm^−/−^ cell lines were generated from p24 ELISA values obtained at day 6 for LEDGIN 7 concentrations of 0 µM, 0.06 µM, 0.2 µM, 0.7 µM, 3.5 µM, 8.9 µM, 31 µM. Data are fitted to a sigmoidal dose-response (variable slope) curve, from which IC_50_ values were calculated (Table S1). (F) In analogy with (E), p24 values obtained at day 6 for RAL were plotted to enable determination of IC_50_. RAL was used in concentrations of 0 µM, 0.002 µM, 0.007 µM, 0.02 µM, 0.08 µM and 0.3 µM.

Recently, we reported a new class of antiretrovirals termed LEDGINs that bind to the LEDGF/p75 binding pocket of HIV-1 IN and block HIV-1 integration and replication in cell culture [25]. We assayed their activity in the LEDGF/p75 KO cells. We challenged Nalm^+/+^ and Nalm^+/c^ cells together with Nalm^−/−^ cells with the laboratory strain HIV_IIIb_ in the presence of different concentrations of LEDGIN 7 [25]. LEDGIN 7 blocked HIV-1 replication in all cell lines in a concentration dependent manner (Figure 5A and 5C). Similar data were obtained with the laboratory strain HIV_NL4.3_ (Figure S5A). The toxicity profile in Nalm-6 cells corresponded to that elaborated previously in MT4 cells [25]. No significant toxicity was observed in the concentrations used (data not shown). Of note, LEDGINs were also active against HIV harvested from LEDGF/p75 KO cells (HIV^−/−^, data not shown). RAL served as a positive control, demonstrating equal inhibition of HIV-1 replication in the different cell lines (Figure 5B and 5D). Dose response curves (Figure 5E and 5F) enabled determination of IC_50_ values, listed in Table S1.

### LEDGINs also disrupt the interaction of HIV-1 IN with HRP-2

**Figure 6:**
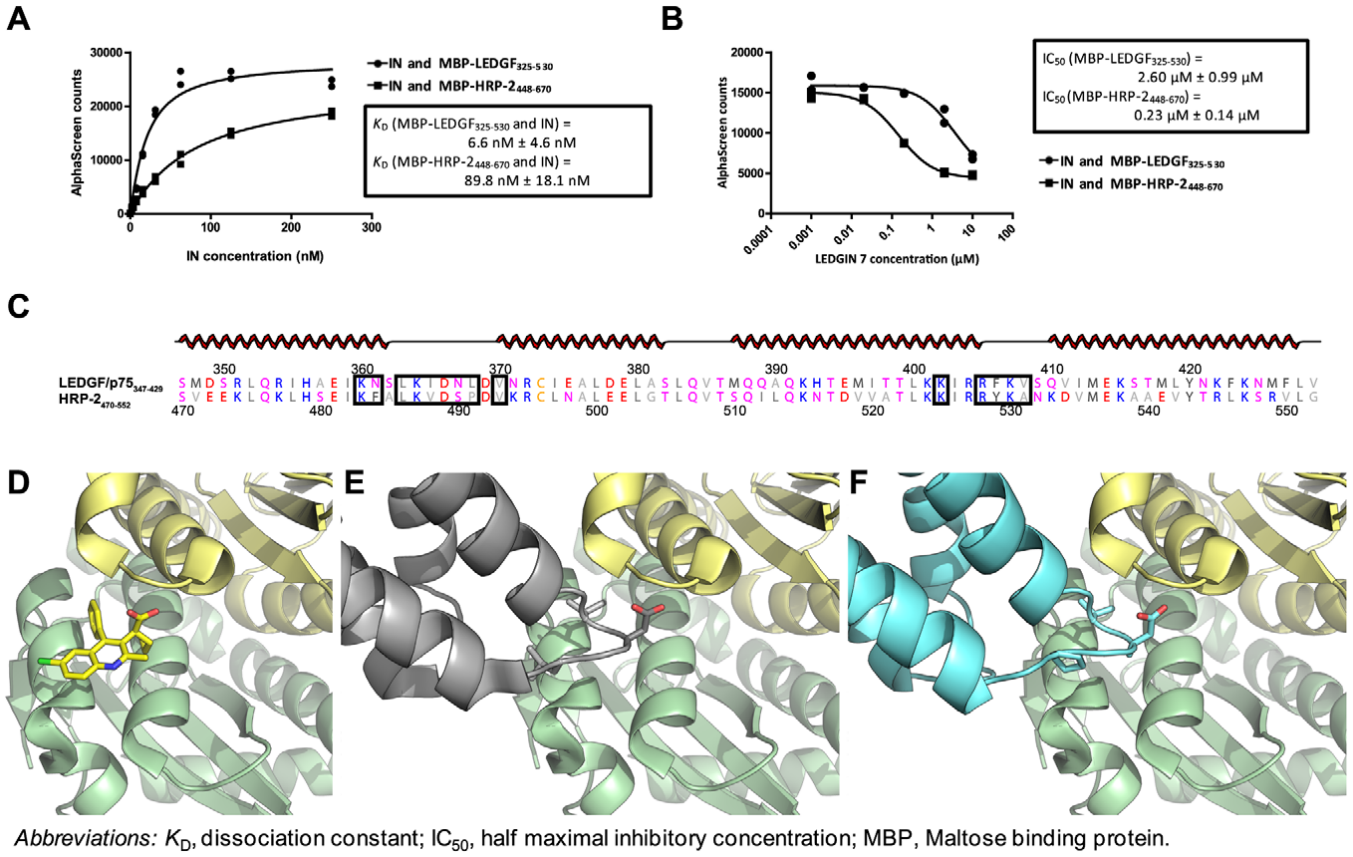
LEDGINs block the interaction of HIV-1 IN with the IBD of either LEDGF/p75 or HRP-2. (A,B) Interactions between His_6_-tagged IN and MBP-LEDGF_325–530_ or MBP-HRP-2_448–670_ were assessed using AlphaScreen technology. (A) 10 nM MBP-LEDGF_325–530_ (circles) or MBP-HRP-2_448–670_ (squares) was incubated with various concentrations of IN (2-fold dilutions from 500 to 1.9 nM). Anti-MBP donor and Ni^2+^-chelate acceptor beads were added and the AlphaScreen signal was read on an EnVision Multilabel reader. Boxed data show average apparent *K*_D_ values ± S.E.M. for the IN-MBP-LEDGF_325–530_ or IN-MBP-HRP-2_448–670_ interaction as determined from four independent experiments, each performed in duplicate. A representative experiment is shown. (B) MBP-LEDGF_325–530_ or MBP-HRP-2_448–670_ (10 nM) was incubated with IN (500 nM) and various concentrations of LEDGIN 7 (10, 2, 0.2, 0.02 and 0 µM, the latter plotted as 0.001 µM). Again, beads were added and the plate was read as described. Boxed data show IC_50_ values of LEDGIN 7 on the interaction of IN with MBP-LEDGF_325–530_ or MBP-HRP-2_448–670_. Average IC_50_ values ± standard deviations were derived from three independent experiments each performed in duplicate. A representative experiment is shown. In (C–F) the homology of LEDGF/p75_IBD_ and HRP-2_IBD_ and their interaction with IN is shown. (C) Alignment of LEDGF/p75_347–429_ and HRP-2_470–552_. Amino acids are colored by type and secondary structure is depicted on top (α-helices in red). Residues involved in the interaction with IN catalytic core domain are boxed. Alignments were generated with ClustalW [63] and further visualized in ALINE [64]. (D–F) Cartoon representations of the IN catalytic core domain (CCD) dimer (pale green and pale yellow) with (D) LEDGIN 6 (sticks, colored by atom), (E) LEDGF/p75_IBD_ (gray) or (F) HRP-2_IBD_ (cyan). Side chains of the crucial amino acids, mimicked by LEDGINs, i.e. residues I365, D366, L368 of LEDGF/p75, and corresponding HRP-2 residues V488, D489 and P491, are shown in sticks colored by atom. Structures of LEDGF/p75_IBD_ in complex with IN CCD dimer were taken from PDB ID: 2B4J [65]. HRP-2_IBD_ homology models were built with MODELLER [66], based upon the same LEDGF/p75_IBD_ – IN CCD dimer structure. The structure of the LEDGIN 6 – IN CCD dimer complex was taken from PDB ID: 3LPU [25]. All structures were visualized in PyMol (DeLano Scientific LLC, Palo Alto, USA).

We have shown that residual replication of HIV-1 laboratory strains in LEDGF/p75 KO cells is predominantly mediated by HRP-2 and that LEDGINs block residual HIV-1 replication in KO cells. This can be explained by allosteric inhibition of LEDGINs or by the fact that binding of LEDGINs to the IN-surface also impedes the interaction with HRP-2 or a combination of both. We evaluated whether LEDGINs inhibit the HRP-2-IN interaction in an AlphaScreen assay. Since IN binds HRP-2 via its IBD (aa 470–593) [12] *in vitro*, we measured the interaction between recombinant HIV-1 IN and the C-terminal part of HRP-2 (aa 448–670). We generated maltose binding protein (MBP) tagged fusions containing either the C-terminal end of LEDGF/p75 (aa 325–530) or HRP-2 (aa 448–670). These recombinant proteins, MBP-LEDGF/p75_325–530_ and MBP-HRP-2_448–670_, bound to His_6_-IN with apparent *K*_D_'s of 6.6 nM (±4.6 nM) or 89.8 nM (±18.1 nM), respectively (Figure 6A). In line with previous observations [25], LEDGINs inhibited the IN-LEDGF_325–530_ interaction (Figure 6B; IC_50_ = 2.60±0.99 µM). LEDGINs also inhibited the IN-HRP-2_448–670_ interaction, albeit with a 10-fold lower IC_50_ (Figure 6B; IC_50_ = 0.23±0.14 µM). This 10-fold increased potency for LEDGIN 7 to block interaction of IN with MBP-HRP-2_448–670_ compared to MBP-LEDGF_325–530_ correlates well with the 13-fold lower affinity of MBP-HRP-2_448–670_ for IN, as shown in Figure 6A.

Next, we evaluated whether LEDGINs remain active in LEDGF/p75 KO HRP-2 KD cells. The residual HIV-1 replication was sensitive to inhibition by LEDGINs (Figure S5E).

## Discussion

Since the identification of LEDGF/p75 as a binding partner of HIV-1 IN in 2003 [1], we and other groups have demonstrated its importance for HIV-1 replication [3]–[7], [10], [11], [34], [35]. Our current understanding of the mechanism of action proposes LEDGF/p75 to act as a molecular tether between the lentiviral preintegration complex and the host cell chromatin; the chromatin reading capacity of LEDGF/p75 thereby determines integration site distribution [2], [11], [17], [18]. Given the methodological restrictions associated with the RNAi and mouse KO studies of the past, we decided to investigate the role of LEDGF/p75 in HIV-1 replication by generating a human somatic LEDGF/p75 KO cell line. A second rationale for this study follows the recent development of LEDGINs, small molecules that efficiently target the interaction between HIV-1 IN and LEDGF/p75 by interaction with the LEDGF/p75 binding pocket in HIV-1 IN [25]. Since LEDGINs block HIV-1 replication, the interest in the question whether or not LEDGF/p75 is essential for viral replication was revived.

Our studies demonstrate that residual HIV-1 replication in LEDGF/p75 KO cells can be observed using laboratory-adapted HIV-1 strains (Figure 2A). These observations are reminiscent to data obtained in LEDGF/p75 KD cell lines [5], [6], [20], although important differences can be noticed. First, when clinical HIV-1 isolates were used, we observed sterilizing infections in LEDGF/p75 KO cells (Figure 2B and 2C). Sterilizing infection has never been reported with RNAi mediated LEDGF/p75 KD. Although the effect might be in part explained by a lower infectivity of these clinical isolates, it emphasizes the importance of LEDGF/p75 for HIV-1 replication. In addition, LEDGF/p75 KO results in a more pronounced shift of HIV-1 integration out of RefSeq genes when compared to control cells (25.7% difference when comparing LEDGF/p75 KO to control cells; Table S3, see column 8), whereas integration in LEDGF/p75 KD cells was only moderately affected (1.6–8.4% compared to control cells, Table S3, see column 8) [2].

A next application of our KO cell line was the investigation of the role of HRP-2 in HIV-1 replication. The cellular function of HRP-2 is currently unknown. Like LEDGF/p75, HRP-2 contains a PWWP domain at its N-terminus [12], [22], [36], [37] and a basic C-terminus, that harbors an IBD-like domain. GST pull-downs showed that the homologous IBD region in HRP-2 (amino acids 470–593) interacts with IN [12]. Vanegas and colleagues reported earlier that HRP-2 overexpression relocated HIV-1 IN from the cytoplasm to the nucleus in LEDGF/p75 depleted cells [23]. Although HRP-2 was investigated previously as a potential alternative for LEDGF/p75, no effect in multiple round HIV-1 replication was observed after HRP-2 KD alone or in combination with LEDGF/p75 KD [6], [20], [24]. However, these observations may have been obscured by the remaining LEDGF/p75 after incomplete RNAi mediated KD. Therefore we revisited the mechanism of residual replication of HIV-1 laboratory strains in LEDGF/p75 KO cell lines. We demonstrate that both single round transduction and multiple round replication is additionally hampered upon HRP-2 KD in LEDGF/p75 KO cells. HIV-1 engages HRP-2 as an alternative for LEDGF/p75, but this low affinity IN binding partner (Figure 6A) can only substitute for LEDGF/p75 after depletion of the latter (Figure 3, 4 and S4), suggesting a dominant role for LEDGF/p75 over HRP-2. Several reasons can be proposed. Cherepanov et al. [12] demonstrated that considerably less IN could be co-immunoprecipitated by HRP-2 than LEDGF/p75, implying that the IN–HRP-2 interaction is weaker than the IN-LEDGF/p75 interaction. In line with these observations, Vanegas et al. reported that Flag-LEDGF/p75 but not Flag-HRP-2 co-immunoprecipitated IN from cell lysates [23]. Here we demonstrate using AlphaScreen technology that the IBD containing C-terminal end of HRP-2 has an approximately 13-fold lower affinity for HIV-1 IN than the corresponding part in LEDGF/p75 (Figure 6A). Next, LEDGF/p75 demonstrates a speckled nuclear localization pattern and binds to mitotic chromatin. Vanegas et al. demonstrated that contrary to LEDGF/p75, HRP-2 does not bind to mitotic chromatin [23] questioning its role as a chromatin-tethering molecule. However, since LEDGF/p75 KD also affects viral replication in non-dividing macrophages [20], the binding capacity of LEDGF/p75 to condensed mitotic chromatin might not be relevant for HIV-1 replication.

The preference of HIV-1 to integration in genes [38] is reduced upon LEDGF/p75 KO corroborating previous observations in LEDGF/p75 KD cells [2], [11], [17], [18] and underscoring LEDGF/p75 as the major targeting factor for HIV-1 integration. In line with this tethering role for LEDGF/p75, chimeras carrying alternative chromatin binding motifs fused to IBD could retarget HIV-1 integration [18], [39], [40]. In addition, De Rijck et al. [41] demonstrated that the LEDGF/p75 chromatin binding mirrors HIV-1 integration site distribution. HIV-1 integration in RefSeq genes remained significantly different from MRC throughout (*P*<0.0001) and more directed towards CpG islands in LEDGF/p75 KO cells. Both observations support the idea of an alternative targeting mechanism for HIV-1 acting in the absence of LEDGF/p75. Since additional HRP-2 KD resulted in an additional 2-fold reduction in integrated copies compared to LEDGF/p75 depletion, HRP-2 is a candidate. The integration site distribution pattern of HIV-1 derived vectors remained unaltered after additional HRP-2 KD in LEDGF/p75 KD HEK293T cells [2], but LEDGF/p75 depletion may have been insufficient in those experiments.

Apart from LEDGF/p75 and HRP-2, no other human protein contains a PWWP-domain in conjunction with an IBD. However, other proteins or protein complexes could take over the tethering activity in the absence of LEDGF/p75 and HRP-2 by combining an IBD-like domain with a chromatin-binding function. The IBD belongs to a family exemplified by the Transcription Factor IIS (TFIIS) N-terminal domain (InterPro IPR017923 TFIIS_N) ([12] and based on an updated search using the HHpred algorithm [42], [43]). Sequence comparison of the respective predicted IN-binding loops of these domains, suggests it is however unlikely that IN binds to these IBD-like proteins as it does to the IBD of LEDGF/p75 or HRP-2 (data not shown). Therefore the residual HIV-1 replication observed in the LEDGF/p75 KO HRP-2 KD cells may 1) still be HRP-2 mediated since the KD of HRP-2 is not complete, 2) be mediated by an unknown third cellular cofactor or complex, or 3) occur independently from cellular cofactors.

The question remains whether HRP-2 is of any importance for HIV infection in patients? The HRP-2 phenotype only becomes evident *in vitro* using laboratory strains and upon strong depletion or KO of LEDGF/p75. Taking into account the lower affinity of HRP-2 for HIV-1 IN, interaction likely only takes place in the complete absence of LEDGF/p75. The LEDGF/p75_IBD_ is highly conserved within humans and across species [12]. Only a few SNPs have been identified [44]. Although relative LEDGF/p75 and HRP-2 expression levels still need to be verified in relevant human cells, to date there is no evidence for LEDGF/p75 depletion in humans and a substituting role of HRP-2 in HIV-1 infection.

Previous reports demonstrated a moderate increase in 2-LTR circles upon LEDGF/p75 KD [5], [6], whereas 2-LTR circles were not significantly different in LEDGF KO MEFs [11]. In this study, we observed no clear difference in the number of 2-LTR circles upon LEDGF/p75 KO. Possibly, the complete absence of LEDGF/p75 affects other steps besides integration that might result in reduced nuclear import and circle formation. Alternatively, cellular pathways involved in 2-LTR formation may be affected. Opposing effects on circle formation by reduced import and reduced integration may finally result in an equal 2-LTR circle number. Alternatively, the sensitivity of 2-LTR circle quantification may be too low to detect a small increase.

In the last part of the manuscript we demonstrate that LEDGINs block the residual replication observed in LEDGF/p75 KO cell lines (Figure 5C) and block the interaction *in vitro* between HRP-2_IBD_ and IN (Figure 6B). Figure 6D illustrates how LEDGINs fit in the pocket at the IN core dimer interface. LEDGINs block the interaction with two interhelical loops of the IBDs of LEDGF/p75 (Figure 6E) or HRP-2 (Figure 6F). The inhibition of the interaction with HRP-2 can explain why residual replication of HIV-1 in LEDGF/p75 KO cells is still sensitive to LEDGINs. Since LEDGF/p75 has been reported to act as an allosteric modulator of the IN activity *in vitro* [1], [12], [45], [46], it is plausible that inhibition of the LEDGF/p75-IN interaction not only interferes with its function as a molecular tether but also results in an allosteric inhibition of IN activity. In fact, inhibition of *in vitro* IN activity in the absence of LEDGF/p75 by potent LEDGINs has been reported [25]. The allosteric mode of inhibition by LEDGINs can as well explain inhibition of HIV-1 replication in LEDGF/p75 KO HRP-2 KD cells [25]. *In vivo* both mechanisms are intrinsically coupled. LEDGINs compete with LEDGF/p75 as a molecular tether and at the same time interfere with integrase activities probably by affecting conformational flexibility in the intasome. Whereas transdominant inhibition of HIV-1 replication by IBD overexpression [4], [35] presumably also acts through this dual mechanism [46], RNAi-mediated depletion of LEDGF/p75 likely only affects tethering and/or targeting. We should however be cautious to translate the results in KO cells to human patients. Since no individuals without functional LEDGF/p75 expression have been documented, LEDGINs will always have to compete with LEDGF/p75 for the IN binding pocket to inhibit integration.

Somatic KO cell lines are cumbersome to generate. This is why few studies used this technology to study the role of cellular cofactors in virus replication. Previously, the role of cyclophilin A in HIV replication was confirmed in a human somatic KO cell line [47] as well as the roles of *CBF1* [48] and *TB7* [49] in Epstein-Barr virus replication. Our work supports the value of generating human KO cell lines for cofactor validation and drug discovery in general.

## Materials and Methods

### Cells and culture

Nalm-6 cells, SupT1 cells, obtained from the ATCC (Manassas, VA) and PM1 cells, a kind gift from Dorothee von Laer (Innsbruck Medical University, Innsbruck, Austria), were maintained in RPMI 1640 – GlutaMAX-I (Invitrogen, Merelbeke, Belgium) supplemented with 8% heat-inactivated fetal calf serum (FCS; Harlan Sera-Lab Ltd.) and 50 µg/ml gentamycin (Gibco, Invitrogen). HEK293T cells, obtained from O. Danos (Genethon, Evry, France), and HeLaP4 cells, a kind gift from Pierre Charneau, Institut Pasteur, Paris, France, were grown in DMEM (Invitrogen) supplemented with 5% FCS, 50 µg/ml gentamycin and 0.5 mg/ml geneticin (Invitrogen). All cells were grown in a humidified atmosphere with 5% CO2 at 37°C.

### Growth kinetics

For growth curve analysis, Nalm-6 cells were seeded at 100,000 in 5 ml of corresponding medium and HeLaP4 cells at 200,000 per well in a 6-well plate. Cell growth was followed on consecutive days by cytometry (Coulter Z1, Beckmann Coulter). Experiments were performed in triplicate.

### Plasmids

The HIV-based lentiviral transfer plasmid pCHMWS_CD4_IRES_Bsd encodes the CD4 receptor driven by a human early cytomegalovirus (CMV) promoter followed by an EMCV internal ribosomal entry site (IRES) and a blasticidin resistance cassette (Bsd). The plasmid was generated by PCR amplification of human CD4 from a T-cell cDNA library using CD4-Fwd and CD4-Rev, followed by digestion with BamHI and XbaI, and cloning into pCHMWS_LEDGF_BC_IRES_Bsd [18], digested with BamHI and SpeI.

The lentiviral transfer plasmids for miRNA-based KD were generated based on miRNA-R30 as previously described [50], [51] (Table S2). For HRP-2 KD, miR HRP-2 was adapted from the sequence validated previously [6]. As negative controls a non-functional, scrambled miRNA30-based short-hairpin sequence (miR scrambled) and a functional, short-hairpin sequence targeting the monomeric red fluorescent protein from *Discosoma corallimorpharia*, DsRed (miR DsRed) were designed [33]. PCR fragments were introduced into the XhoI–BamHI sites from a modified pN3-eGFP plasmid (Clontech, Saint Quentin Yuelines, France) duplicated and cloned into the XhoI-KpnI sites at the 3′ end of the enhanced green fluorescent protein (eGFP) reporter cDNA, driven by a Spleen focus forming virus LTR (SFFV) promoter, resulting in pCSMWS_eGFP_miR_HRP2 and pCSMWS_eGFP_miR_scrambled. To generate pCSMWS_Zeo_miR_HRP2, the zeocin resistance cassette (Zeo) was amplified with primers Zeo-Fwd and Zeo-Rev from pBUD (Invitogen), digested with NheI-Pfl23II and inserted into the XbaI–Pfl23II digested pCSMWS_eGFP_miR_HRP2 plasmid. To generate pCSMWS_Zeo_miR_DsRed, miR_DsRed was cloned into the XhoI/KpnI digested pCSMWS_Zeo_miR_HRP2 plasmid. To generate pCSMWS_Hygro_miR_HRP2, the hygromycin B resistance cassette (Hygro) was amplified using Hygro-Fwd and Hygro-Rev as primers and pBud (Invitrogen) as a template. The resulting products were digested ClaI-XhoI and ligated into pCSMWS_Zeo_miR_HRP2.

For bacterial expression of C-terminal His_6_-tagged HIV-1 IN and MBP-tagged-LEDGF_325–530_, the plasmids pKBIN6H [10] and pMBP-Δ325 [4] were used, respectively. To construct pMBP-HRP-2_448–670_, the sequence corresponding to aa 448 to 670 of HRP-2 was PCR amplified with primers HRP2-Fwd, and HRP2-Rev, using p3xFlagHRP-2 (a kind gift from E. Poeschla) as a template. The resulting products were digested and ligated into pMAL-c2E (New England Biolabs Inc., USA). The integrity of all plasmids was confirmed by DNA sequencing.

### Retroviral vector production and transduction

LV production was performed as described earlier [18], [52]. Briefly, vesicular stomatitis virus glycoprotein (VSV-G) pseudotyped lentiviral vector particles were produced by PEI transfection in HEK293T cells using the different transfer plasmids. Single round HIV_NL4.3ΔNefΔEnv_fLuc (HIV-fLuc) virus was prepared by co-transfection of HEK293T cells with pNL4-3.LucR–E– (pHIV-fLuc, National Institutes of Health AIDS Research and Reference Reagent Program) and pMD.G, that codes for VSV-G.

For lentiviral transduction experiments, Nalm-6 cells were typically plated at 150,000 cells per well in a 96-well plate and transduced overnight. After 72 hrs, 50% of cells were reseeded for luciferase expression quantification and/or FACS analysis. The remainder was cultured for quantitative PCR and integration site analysis during at least 10 days to eliminate non-integrated DNA. HeLaP4 cells were plated at 20,000 cells per well in a 96-well plate and transduced overnight. After 72 hrs, 50% of cells were reseeded for luciferase quantification. The remainder was cultured for quantitative PCR or integration site analysis as described for Nalm-6 cells.

### *PSIP1* KO targeting plasmids

Targeting plasmids for generation of *PSIP1* KO were designed and cloned as described previously [27], [53] utilizing the MultiSite Gateway System (Invitrogen) as described [54]. Briefly, a 2.3 and a 2.0 kb fragment for the left and right arms of the targeting plasmids, respectively, were amplified by genomic PCR using primers LEDGF/p75 attB4 and LEDGF/p75 attB1 for the left arm, and primers LEDGF/p75 attB2 and LEDGF/p75 attB3 for the right arm (Table S2). The resulting fragments were cloned into pDONR/P4-P1R and pDONR/P2R-P3 via recombination, resulting in p5′-ENTR-left and p3′-ENTR-right, respectively. The fragments p5′-ENTR-left, p3′-ENTR-right, pDEST DTA-MLS, and pENTR lox-Puro or pENTR lox-Hygro, were then ligated using recombination to generate the final targeting plasmids pTARGET-LEDGF/p75-Hyg and pTARGET-LEDGF/p75-Puro, respectively.

### Generation and validation of LEDGF/p75 KO cell lines

Cell lines were generated as previously described [27]. Briefly, targeting plasmid was transfected with Nucleofector I (Amaxa, Inc., Gaithersburg, MD, USA) using 2×10^6^ Nalm-6 cells and 2 µg of DNA. At 24 hrs after transfection, cells were seeded into 96-well plates at 10^3^ cells per well, in culture medium supplemented with either 0.2 µg/ml puromycin (BD BioSciences, San Jose, CA, USA) or 0.35 mg/ml of hygromycin B (Clontech, Mountain View, CA, USA). After 2–3 weeks, individual drug resistant colonies were propagated and analyzed by genomic PCR using primers A and B or C (Figure 1A), generating a 3272 bp AB-fragment for Nalm^+/c^, Nalm^c/c^, Nalm^−/−^ and a 3123 bp AC-fragment for Nalm^c/c^, Nalm^−/−^ (Figure 1C). Genomic PCR of the targeted region was performed with primers D and E generating a 1624 bp DE-fragment in Nalm^+/+^, Nalm^+/c^ and a 288 bp DE-fragment in Nalm^−/−^ (Figure 1C). Targeting efficiency was calculated as the ratio of the number of cell clones where the LEDGF/p75 allele was disrupted by homologous recombination to the number of drug-resistant cell clones (Figure 1B). Sequencing of the genomic KO region was performed as follows. The PCR fragments obtained after amplification with primers gFB and gRB spanning a 1885 bp region around exon 11–14 in wild-type cells or a 571 bp region in KO cells, followed by nested PCR with primers gFA and gRA resulting in a 1694 bp in wild-type or 380 bp region in KO cells, were cloned into the pCRII-TOPO plasmid (Invitrogen, Merelbeke, Belgium) and sequenced with primers M13-Fwd and M13-Rev (Figure S1A). Total RNA extracted from KO clones (RNeasy 96 kit, Qiagen) was used for cDNA synthesis using oligo-dT primers (High capacity cDNA RT kit, Applied biosystems). Correct recombination was verified at mRNA level for LEDGF/p52, LEDGF/p75 and truncated LEDGF/p75 by PCR on cDNA using primers RNA-A and RNA-B for LEDGF/p52, LEDGF/p75 and LEDGF^KO^, RNA-A and RNA-C for LEDGF/p75 and LEDGF^KO^, RNA-A and RNA-D for LEDGF/p52, resulting in fragments of 245 bp, 1.606 kb or 1.163 kb and 1.011 kb, respectively (Figure S1C). Additionally, the cDNA of the truncated protein was sequence verified (Figure S1A) as follows: a PCR product generated by primers d243 and RNA-C, followed by nested PCR with primers d244 and LEDGF-R-exon15, was cloned into pCRII-TOPO (Invitrogen) and sequenced with M13-Fwd and M13-Rev. Primers are listed in Table S2.

### Generation of stable CD4 expressing Nalm-6 cell lines

Stable CD4 expressing Nalm-6 cell lines were generated by transducing wild-type Nalm^+/+^, Nalm^+/c^ cl 31 and Nalm^−/−^ cl 1 and cl 2, with the lentiviral vector pCHMWS_CD4_IRES_Bsd and subsequent selection with blasticidin (3 µg/ml; Invitrogen, Merelbeke, Belgium). CD4 expression was verified by flow cytometry using R-Phytoerythrin-conjugated mouse anti-human CD4 monoclonal antibody (BD pharmigen) according to the manufacturer's protocol.

### Generation of LEDGF/p75 and HRP-2 KD cell lines

Stable monoclonal LEDGF/p75 KD cells were generated previously [18]. Additional HRP-2 KD was obtained by transduction of HeLaP4 wild-type cells and LEDGF/p75 KD cells with pCSMWS_Zeo_miR_HRP2, pCSMWS_Hygro_miR_HRP2 or pCSMWS_eGFP_miR_HRP2. Transduced cells were selected with zeocin (200 µg/ml) or hygromycin B (200 µg/ml) or by FACS sorting of eGFP positive cells respectively. Control cell lines were generated likewise by transduction with vectors encoding pCSMWS_Zeo_miR_DsRed, pCSMWS_eGFP_IRES_HygroR and pCSMWS_eGFP_miR_scrambled, respectively. Stable PM1 and SupT1 LEDGF/p75 KD cell lines were generated with LV, encoding a miRNA cassette targeting LEDGF/p75 under control of an SFFV promoter (unpublished data). Additional HRP-2 KD and control PM1, SupT1 and Nalm-6 cell lines were generated by transducing the cells with vectors made with pCSMWS_Hygro_miR_HRP2 and pCSMWS_eGFP_IRES_HygroR, respectively.

### Integration site amplification and bioinformatic analysis

Integration sites were amplified by linker-Mediated PCR as described previously [17], [18]. For integration sites to be authentic, sequences needed a best unique hit when aligned to the human genome (hg18 draft) using BLAT. The alignment began within 3 bp of the viral long terminal repeat end, and had >98% sequence identity. Reanalysis of previously obtained integration sites [2], [11], [17] was performed in parallel. Statistical methods are described previously [55]. Integration site counts were compared using a two-tailed Fisher's exact test. Analysis was carried out using Prism 5.0 (GraphPad Software).

### Virus strains

The origin of HIV_NL4.3_ [56] and HIV_IIIb_ has been described [57]. Clinical isolate #1 was obtained through the AIDS research and reference reagent program, Division of AIDS, NIAID, NIH: HIV-1 93TH053 from the UNAIDS network for HIV isolation. Clinical isolate #2 was obtained through the AIDS research and reference reagent program, Division of AIDS, NIAID, NIH: HIV-1 96USSN20 from Drs Ellenberger, P. Sullivan and R.B. Lal [32]. The p24 antigen titer was determined for each virus stock. The MOI was determined using flow cytometry analysis of intracellular p24 antigen 24 hrs after infection in control Nalm^+/c^ cells. Cells were stained with pycoerythrin-anti-p24 (KC57-RD1; Beckman Coulter) using the Fix&Perm (Invivogen) cell fixation and cell permeabilization kit following the manufacturer's protocol.

### HIV-1 infection experiments

HIV-1 infection of Nalm-6 cells was typically performed with 1*10^6^ cells in 5 ml of medium with the indicated virus and MOI. After 6–12 hrs of infection, cells were washed twice with PBS and resuspended in the initial volume of culture medium. Infection of HeLaP4 cells was performed as described previously [18]. HIV-1 replication was monitored by quantifying p24 antigen in the supernatant daily via ELISA (Alliance HIV-1 p24 ELISA kit; Perkin Elmer). Cells were split 1/6 every 5–6 days if experiments exceeded 10 days. PM1 and SupT1 cells were infected at 0.01 pg p24/cell.

### PCR amplification and DNA sequencing of the coding regions of IN

Proviral DNA extraction of infected cells was performed using the QIAamp blood kit (Qiagen) according to the manufacturer's protocol. PCR amplification and sequencing of IN encoding sequences were done as described previously [58].

### Antiviral agents

Zidovudine (AZT) and ritonavir (RIT) were purchased and raltegravir (MK518) was kindly provided by Tibotec (Mechelen, Belgium). LEDGIN 7 was synthesized as described [25].

### Luciferase activity assay

Cells harvested from a 96-well plate were lysed with 50 µl lysis buffer (50 mmol/l Tris pH 7.5, 200 mmol/l NaCl, 0.2% NP40, 10% glycerol). The lysate was assayed according to the manufacturer's protocol (ONE-Glow; Promega, Madison, WI). Luciferase activity was normalized for total protein (BCA; Pierce, Rockford, IL). All conditions were run at least in triplicate in each experiment.

### Transfection of plasmid DNA in HeLaP4 cells

HeLaP4 cells were transfected with pHIV-fLuc using Lipofectamine 2000 (Invitrogen, Merelbeke, Belgium) according to the manufacturer's protocol with minor modifications. Briefly, 70,000 cells were seeded in a 96 well plate and transfected after one day with a mixture of 333 ng DNA and 0.66 µl Lipofetamine 2000 for 4 hrs and washed afterwards twice with PBS. 48 hrs post transfection cells were harvested for luciferase activity quantification.

### Quantitative PCR

Quantification of LEDGF/p75 mRNA levels was performed as described previously [18]. Similar settings were used to determine HRP-2 mRNA levels. HRP-2 primer/probe set: HRP2 s4, HRP2 as4 and HRP2 probe. In all cases, RNaseP was used as endogenous house-keeping control (TaqMan RNaseP Control Reagent; Applied Biosystems). All samples were run in triplicate for 3 minutes at 95°C followed by 50 cycles of 10 seconds at 95°C and 30 seconds at 55°C. Data were analyzed with iQ5 Optical System Software (BioRad, Nazareth, Belgium). To quantify the different HIV-1 DNA species qPCR for total viral DNA, 2-LTR circles and integrated copies was performed as described [59], [60], with minor modifications. Nalm-6 cells were seeded one day prior to infection at 2*10^5^ cells per ml. After 4 hrs of incubation with HIV, medium was replaced by RPMI containing 10% FCS. Quantification of proviral copies as shown in Figure 3G was performed accordingly, only RIT (at 50 times IC_50_) was added to the culture medium after the washing step to ensure only a single replication cycle could take place and genomic DNA was isolated after 5 days to dilute all non-integrated forms. Non-infected cells were incubated in parallel. To quantify the number of integrated copies as shown in Figure S4H, cells were cultured for 10 days in medium containing RIT and AZT both at 25 times IC_50_ following day 39, 45 or 48, before harvesting genomic DNA. Quantitative *Alu*-PCR for quantification of proviral copies was done in two steps [60]. The first phase amplifies from *Alu* sequences to U3 sequences absent in self-inactivating (U3-deleted) HIV-1 vectors using 400 nM *Alu*SINIIfwd, 400 nM q*Alu*Rout_SB704. Amplification conditions were 95°C for 30 sec, 60°C for 40 sec, 72°C for 1 min 30 sec, ×13 cycles. The second phase amplifies a nested product using 300 nM sense primer Q-*Alu*-F-in, 300 nM antisense Q-*Alu*-R-in and 200 nM *Alu*-probe. PCR conditions were 95°C for 10 sec, 55°C for 30 sec, ×50 cycles.

### Southern blot analysis

Southern blot analysis was performed as described previously in [61], [62]. Briefly, genomic DNA was digested with BamHI, separated by electrophoresis on a 0.7% agarose gel and blotted on positively charged nylon membranes (Biodyne B; Pall Corp., Pensacola, FL, USA). The probe covered a 1024 bp genomic region around exon 10 of *PSIP1* and was amplified by PCR using LPROBE-Fwd and LPROBE-Rev, and labeled with α-^32^P-dCTP (Megaprime DNA labeling system, GE Healthcare, USA). Signals were detected using autoradiography.

### Western blotting

Western blotting was performed as described previously [5]. Briefly, cellular extracts were separated by sodium dodecyl sulfate–polyacrylamide gel electrophoresis. LEDGF was detected using a purified IgG1 mouse anti-human LEDGF monoclonal antibody (mAb, C26) (BD Biosciences Pharmingen, San Diego, CA). Equal loading was verified with β-tubulin (T-4026; Sigma-Aldrich, St Louis, MO). Visualisation was performed by chemiluminescence (ECL+; Amersham Biosciences, Uppsala, Sweden).

### Production and purification of recombinant proteins

Recombinant HIV-1 IN containing a C-terminal His_6_ tag was purified as described previously [10]. LEDGF_325–530_ and HRP-2_448–670_ fragments were expressed in *E. coli* as maltose binding protein (MBP) fusions. The purification of pMBP-LEDGF_325–530_ from BL21(DE3) bacterial cells was done as described previously [4]. For purification, cells were resuspended in lysis buffer (50 mM Tris-HCl, pH 7.2, 500 mM NaCl, 5 mM dithiothreitol, 1 mM EDTA, 0.2 mM phenylmethylsulfonyl fluoride, 0.1 U/ml DNase). After complete lysis by ultrasonication, the supernatant was cleared by centrifugation and recombinant proteins were bound to amylose resin (New England Biolabs Inc, United Kingdom). The resin was washed with 20 bed volumes wash buffer (50 mM Tris-HCl, pH 7.2, 500 mM NaCl, 5 mM dithiothreitol), and the MBP-tagged proteins were eluted in 1 ml fractions wash buffer supplemented with 10 mM maltose. The fractions were analyzed by sodium dodecyl sulfate-polyacrylamide gel electrophoresis for protein content, pooled, and concentrated by dialysis (overnight, 4°C) against storage buffer (50 mM Tris-HCl, pH 7.2, 500 mM NaCl, 50% (vol/vol) glycerol). All protein concentrations were measured using the Bradford assay (Bio-Rad).

### AlphaScreen

AlphaScreen measurements were performed in a total volume of 25 µL in 384-well Optiwell microtiter plates (PerkinElmer). All components were diluted to their desired concentrations in assay buffer (25 mM Tris-HCl pH 7.4, 150 mM NaCl, 1 mM MgCl_2_, 0.1% Tween-20 and 0.1% BSA). Anti-MBP coated donor beads were generated by dialyzing biotin-labelled anti-MBP (Vector Laboratories) to the assay buffer and incubating 10 nM of this antibody with the desired amount of Streptavidin donor beads (PerkinElmer) for 1 h at room temperature. For the *K*_D_ determinations, HIV-1 IN-His_6_ was titrated against a background of 10 nM MBP-LEDGF_325–530_ or MBP-HRP-2_448–670._ This amount provided minimal binding curve perturbation while maintaining a good signal-to-noise ratio. When performing IC_50_ determinations, LEDGIN 7 was titrated against a background of 500 nM IN-His_6_ and 10 nM MBP-LEDGF_325–530_ or MBP-HRP-2_448–670_. After addition of the proteins and/or compounds, the plate was incubated for 1 h at 4°C and 20 µg/mL anti-MBP donor and Ni^2+^-chelate acceptor beads (PerkinElmer) were admixed, bringing the final volume to 25 µL. After 1 h of incubation at RT, protected from light, the plate was read on an EnVision Multilabel Reader in AlphaScreen mode (PerkinElmer). Results were analyzed in Prism 5.0 (GraphPad software) after non-linear regression with the appropriate equations: one-site specific binding, taking ligand depletion into account for the *K*_D_ measurements and sigmoidal dose-response with variable slope for the IC_50_ determination.

### Accession numbers

The Genbank (http://www.ncbi.nlm.nih.gov/genbank) accession numbers for the proteins discussed in this paper are LEDGF/p52 (NM_021144.3), LEDGF/p75 (NM_033222.3) and HRP-2 (NM_032631.2).

## Supporting Information

Figure S1 **Characterization of LEDGF/p75 KO cells.** (A) Scheme of LEDGF/p75, LEDGF/p52 and the truncated variant of LEDGF/p75 after proficient KO (LEDGF^KO^). Different domains of LEDGF are shown: PWWP-domain, nuclear localization signal (NLS), AT-hook like domains (AT-hooks) and the IBD. The sequencing product of the mRNA of LEDGF^KO^ in the KO clones reveals the predicted frame shift after joining of exon 10 and 15, leading towards a premature stop codon. (B) Scheme of mRNA transcripts of LEDGF/p52 and LEDGF/p75. The KO region is indicated with a horizontal red bar. (C) Analysis of mRNA with reverse transcriptase PCR (RT-PCR), indicated primers are depicted in B (see Table S2). (D) Expression levels of LEDGF/p75 mRNA was evaluated with quantitative reverse transcriptase PCR (qRT-PCR), normalized for RNaseP expression levels and depicted relative to wild-type levels. Primer E and F used are depicted in B. Average ± standard deviations are shown from experiments performed at least in triplicate. (E–F) Analysis of HIV-1 DNA species in Nalm^+/c^ and Nalm^−/−^ cell lines at different time points after infection with single round HIV-fLuc. (E) Relative number of total viral cDNA transcripts and (F) 2-LTR circles. Experiments were performed in duplicate and analyzed with qPCR in triplicate. Copy number ratio normalized to RNaseP of a representative experiment is shown with standard deviations. (G–H) HIV-1 integration site distribution analysis. Relative number of experimentally derived HIV-1 integration events in genes according to the Ensembl (G) and UniGene (H) annotation, versus computationally generated matched random controls (MRC). (I–J) Integration site consensus at sequences flanking HIV-1 proviruses in control and LEDGF/p75 KO Nalm-6 cells is shown. (I) HIV-1 in Nalm^+/c^, (J) HIV-1 in Nalm^−/−^ (data from Nalm^−/−^ cl 1 and 2 were pooled). The diagrams were generated using the WebLOGO program (weblogo.berkeley.edu/logo.cgi). The y-axis indicates bits of information with a perfect conservation of a base scoring as two bits.
(EPS)

Figure S2 **Characterization of spreading infection in LEDGF/p75 KO cells.** (A) Control Nalm^+/+^ and Nalm^+/c^ and KO Nalm^−/−^ cl 1 and cl 2 cell lines, were challenged with a laboratory strain HIV_NL4.3_ virus. Cells were infected at different MOI as indicated in the graph. (B) Control Nalm^+/c^ and Nalm^−/−^ cl 2 cells were challenged with the laboratory strain HIV_NL4.3_. Cells were washed after 8 hrs (arrow) and resuspended in medium with or without inhibitors. The gray line above the chart indicates the duration of treatment. AZT and RIT were used at 50 times IC_50_. Mean with standard deviations of experiments performed in duplicate are shown. Replication was monitored by measuring the p24 content in the supernatant. (C) Nalm^+/c^ and KO Nalm^−/−^ cl 1 cells were challenged with HIV^+/c^ (C, left panel) and HIV^−/−^ (C, right panel). Values from three independent experiments were each normalized to the peak p24 amount, consistently observed in Nalm^+/c^ at day 6. Mean relative p24 amount in Nalm^−/−^ at day 15 after challenge with HIV^+/c^ was 7.45%±6.8 and 7.20%±4.0 after challenge with HIV^−/−^.
(EPS)

Figure S3 **Analysis of HIV-1 DNA species in Nalm^+/c^ and Nalm^−/−^ cell lines at different time points after infection with HIV_NL4.3_.** (A) Relative number of total viral DNA, (B) 2-LTR circles and (C) integrated proviral copies (*Alu*-qPCR). Experiments performed in duplicate and analyzed with qPCR in triplicate. Copy number ratio normalized to RNaseP of a representative experiment is shown with standard deviations. RAL was used at 50 times IC_50_ concentration.
(TIF)

Figure S4 **Additional HRP-2 KD blocks residual HIV-1 replication in LEDGF/p75 KD cells.** (A–B) Multiple round HIV-1 replication in HeLaP4 HRP-2 KD cells (wild-type miR HRP-2) and control cell lines (wild-type and wild-type miR control). (A) HRP-2 mRNA levels were determined by qPCR, normalized to RNaseP expression levels and expressed as percentage from wild-type. (B) Cells were challenged with HIV_NL4.3_ and supernatant was harvested for p24 ELISA. Experiments were performed in duplicate; a representative experiment is shown. (C–J) Additional HRP-2 KD (miR LEDGF/miR HRP-2) and control (miR LEDGF/miR control) cell lines were generated from stable LEDGF/p75 KD HeLaP4 cell lines (miR LEDGF). Constructs used to generate the cells are listed below the graph. (C) HRP-2 mRNA expression levels were determined with qPCR, normalized to RNaseP expression levels and expressed as percentage from wild-type. (D) Different cell lines were transduced with HIV-fLuc and luciferase expression was quantified (RLU per µg protein). In (E) the number of integrated copies was determined. Following transduction with HIV-fLuc, cells were grown for an additional 10 days to eliminate non-integrated viral DNA. (F) Different HeLaP4 cell lines were transfected with pHIV-fLuc and luciferase expression was quantified. In panel (G) we challenged the different cell lines with the laboratory strain HIV_NL4.3_. The experiment was continued for 48 days. Supernatant was harvested for p24 ELISA. Experiments were performed in duplicate; a representative experiment is shown. (H) Following multiple round HIV-1 infection as presented in (G), we determined the number of proviral copies by qPCR in miR LEDGF/miR control and miR LEDGF/miR HRP-2 cells on day 39, 45 and 48, when cells were grown for an additional 10 days in the presence of antiretroviral therapy to eliminate non-integrated viral DNA. In (I) we determined late reverse transcripts (Late RT products) at 10 hrs post infection (p.i.) with HIV_NL4.3_ using qPCR, normalized to RNaseP genomic copies. In (J) 2-LTR circles were determined at 24 hrs post infection with HIV_NL4.3_ using qPCR, normalized to RNaseP genomic copies. Average ± standard deviations are shown from experiments performed at least in triplicate.
(TIF)

Figure S5 **Effect of LEDGINs on HIV in LEDGF/p75 KO HRP-2 KD cells.** (A) Control Nalm^+/c^ and KO Nalm^−/−^ cell lines were challenged with the laboratory strain HIV_NL4.3_ in the absence (0 µM) or presence of LEDGIN 7 (31 µM). Experiments were performed in duplicate; average ± standard deviations is shown. (B–E) Stable HRP-2 KD (miR HRP-2) and control (control) Nalm^+/c^ and Nalm^−/−^ cells were challenged with HIV_IIIb_ in the presence of various concentrations of LEDGIN 7 (0 µM, circles; 0.2 µM, squares; 2.5 µM, triangles or 31 µM, triangles pointing downwards). (F) Luciferase activity following HIV-fLuc transduction in the different cell lines is shown. Average ± standard deviations is shown from experiments performed in triplicate. Replication was monitored by measuring the p24 content of the supernatant.
(EPS)

Figure S6 **Cell growth and expression levels of CD4 or CXCR4.** (A) On day zero 100,000 Nalm-6 cells were seeded in triplicate in 5 ml of culture medium. Cells were counted on a daily basis. Average numbers and standard deviations are shown. (B) Different HeLaP4 cell lines were seeded at 200,000 cells in a 6 well format and counted daily. Average and standard deviations of experiments in triplicate are shown. The different vectors used to generate the additional HRP-2 KD-control cel lines are specified within brackets. In (C) the doubling time and 95% confidence interval (95% CI) was calculated after nonlinear regression (exponential growth curve fit). The representing R-square (R^2^) is given. (D) Stable CD4-expressing cell lines selected with blasticidin were generated through transduction with a lentiviral vector (pCHMWS_CD4_IRES_Bsd). Equal expression was verified with flow cytometry using a PE-labeled CD4 antibody. Experiments were performed in triplicate, averages of percentage gated cells times MFI and standard deviation are shown. Nalm^+/+^, Nalm^+/c^, Nalm^−/−^ cl 1 and cl 2 used in this figure represent CD4-expressing descendants from Nalm-6 cell lines as described in Figure 1. (E) Nalm-6 cells express the HIV-1 coreceptor CXCR4 as demonstrated previously. Likewise equal expression was verified with flow cytometry using a PE-labeled CXCR4 antibody.
(EPS)

Figure S7 **Schematic overview of viral vector constructs.** (A) Lentiviral vector construct for CD4 expression. (B–D) Different lentiviral vectors to generate stable HRP-2 KD and control cell lines, ordered pairwise, are shown. *Abbreviations:* IRES, internal ribosomal entry site; Hygro, hygromycin B; HRP-2, Hepatoma derived growth factor related protein 2; Bsd, blasticidin; Zeo, zeocin.
(EPS)

Table S1 **IC_50_ values for LEDGINs (LEDGIN 7) and INSTIs (RAL).** IC_50_ values for LEDGIN 7 and Raltegravir were calculated based on data shown in figure 6. Data were fitted to a sigmoidal dose-response (variable slope) curve, from which IC_50_ values were calculated. Mean and 95% confidence interval are shown.
(DOC)

Table S2 **Overview of primers and probes used.** Primers and probes used throughout the manuscript are shown.
(DOC)

Table S3 **Effect of LEDGF/p75 KD or KO on the frequency of viral integration in genomic features.** Comparison of the HIV integration site distribution pattern elaborated in current and previous publications [2], [11], [17]. Frequency of viral integration in genomic features (integration in RefSeq genes, ±2 kb or ±4 kb around CpG islands) is shown.
(DOC)
