# Correction: LEDGF/p75-Independent HIV-1 Replication Demonstrates a Role for HRP-2 and Remains Sensitive to Inhibition by LEDGINs

**DOI:** 10.1371/journal.ppat.1008894

**Published:** 2020-09-01

**Authors:** Rik Schrijvers, Jan De Rijck, Jonas Demeulemeester, Noritaka Adachi, Sofie Vets, Keshet Ronen, Frauke Christ, Frederic D. Bushman, Zeger Debyser, Rik Gijsbers

There are several errors in Fig 1E and its legend. The top right corner of the blot, corresponding to the upper half of the LEDGF/p75—Nalm^-/-^cl 1 and LEDGF/p75 –Nalm ^-/-^ cl 2 lanes are obscured. No data is obscured.

The splicing of a lane between the Nalm^+/c^ Cl 31 and the Nalm^c/c^ 31 cl 73 lanes in Fig 1E is not indicated. This has now been shown with a black line.

The correct Fig 1, created with the raw blot data and a black line to indicate the spliced lanes, is provided here.

**Fig 1:**
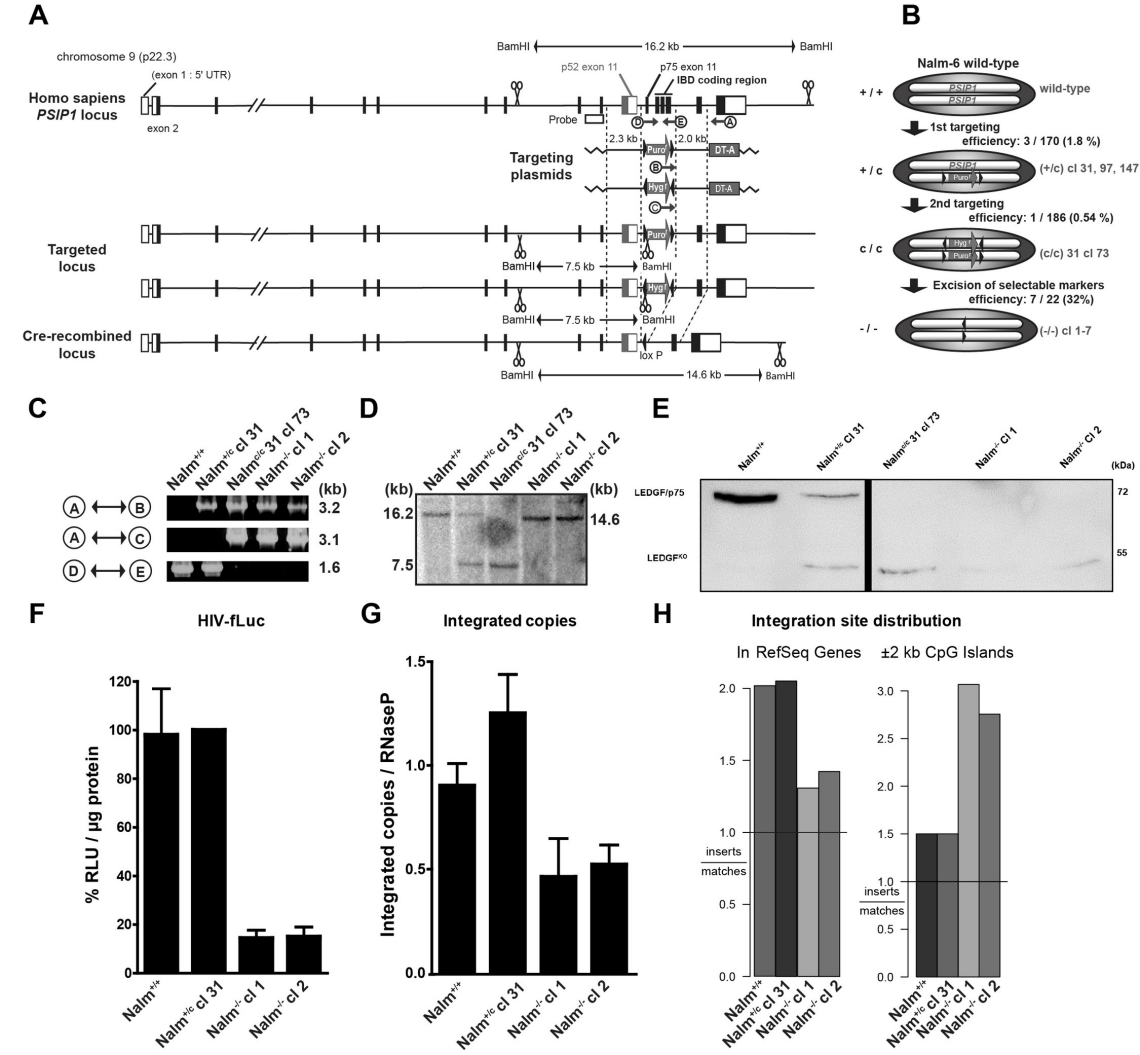
Generation and validation of human LEDGF/p75 KO cell line. (A) Scheme for *PSIP1* gene targeting by homologous recombination. The 2.3 and 2.0 kb arm indicated on the targeting plasmids enable homologous recombination and harbor a puromycin (Puro^r^) or hygromycin B resistance (Hyg^r^) cassette (c) flanked by loxP sites (arrows). Exon 11 of p52 and p75 are indicated separately as well as the IBD coding region. BamHI restriction sites are indicated (scissors). DT-A denotes the gene encoding for Diphteria toxin A. (B) Schematic overview of KO and intermediates: Nalm-6 wild-type (Nalm^+/+^) contains 2 *PSIP1* genes; Nalm^+/c^ clones (cl) 31, 97, 147, contain a puromycin resistance cassette in one allele; Nalm^c/c^ 31 cl 73, contains both a puromycin and a hygromycin B resistance cassette; after CRE-mediated excision cl 1–7 are generated and termed Nalm^−/−^. (C) Genomic PCR on DNA from different clones. Primer binding sites are indicated in panel A (see also Table S2). Indicated bands confirm amplification of a 1.6 kb fragment by primers D and E in full length *PSIP1* as shown in Nalm^+/c^ and Nalm^+/+^, but not in Nalm^c/c^ and Nalm^−/−^ cl 1. (D) Southern blot on genomic DNA after BamHI digestion. Probe and restriction sites are indicated in panel A. Intact *PSIP1* generates a 16.2 kb fragment. After insertion of a resistance cassette a 7.5 kb fragment is generated, after CRE-mediated excision a 14.6 kb fragment is formed indicating KO of a 1.6 kb fragment containing exon 11–14. (E) Western blot analysis for LEDGF protein of whole cell extracts. Marker heights (right), LEDGF/p75 and LEDGF^KO^ are indicated. Nalm-6 wild-type (Nalm^+/+^) contains 2 *PSIP1* alleles; Nalm^+/c^ clone (cl) 31 contains a puromycin resistance cassette (c) in one allele; Nalm^c/c^ 31 cl73 contains a resistance cassette in both *PSIP1* alleles; Nalm−/− cl1 and cl2 have both alleles knocked out. The white line indicates the removal of 1 lane not discussed in this manuscript. (F) Nalm-6 cells were transduced with HIV-fLuc. Luciferase expression is shown as percentage relative light units (RLU) per µg protein as compared to Nalm^+/c^ cl 31. (G) In parallel, the number of integrated proviral copies was evaluated for HIV-fLuc. Following transduction, cells were grown for at least 10 days to eliminate non-integrated viral DNA and analyzed by quantitative PCR. (H) HIV-1 integration site distribution analysis. Left panel shows relative number of experimentally derived HIV-1 integration events in genes according to the RefSeq annotation, versus computationally generated matched random control (MRC). The right panel shows integration events occurring ±2 kb around CpG islands as compared with MRC. Average ± standard deviations are shown from experiments performed at least in triplicate.

## References

[1] Schrijvers R, De Rijck J, Demeulemeester J, Adachi N, Vets S, Ronen K, et al (2012) LEDGF/p75-Independent HIV-1 Replication Demonstrates a Role for HRP-2 and Remains Sensitive to Inhibition by LEDGINs. PLoS Pathog 8(3): e1002558 10.1371/journal.ppat.1002558 22396646 PMC3291655

