# Supplementary figures and images for: LEDGF/p75-Independent HIV-1 Replication Demonstrates a Role for HRP-2 and Remains Sensitive to Inhibition by LEDGINs
Source: PLoS Pathog. 2012 Mar 1;8(3):e1002558. doi: 10.1371/journal.ppat.1002558 (PMC3291655; doi:10.1371/journal.ppat.1002558)

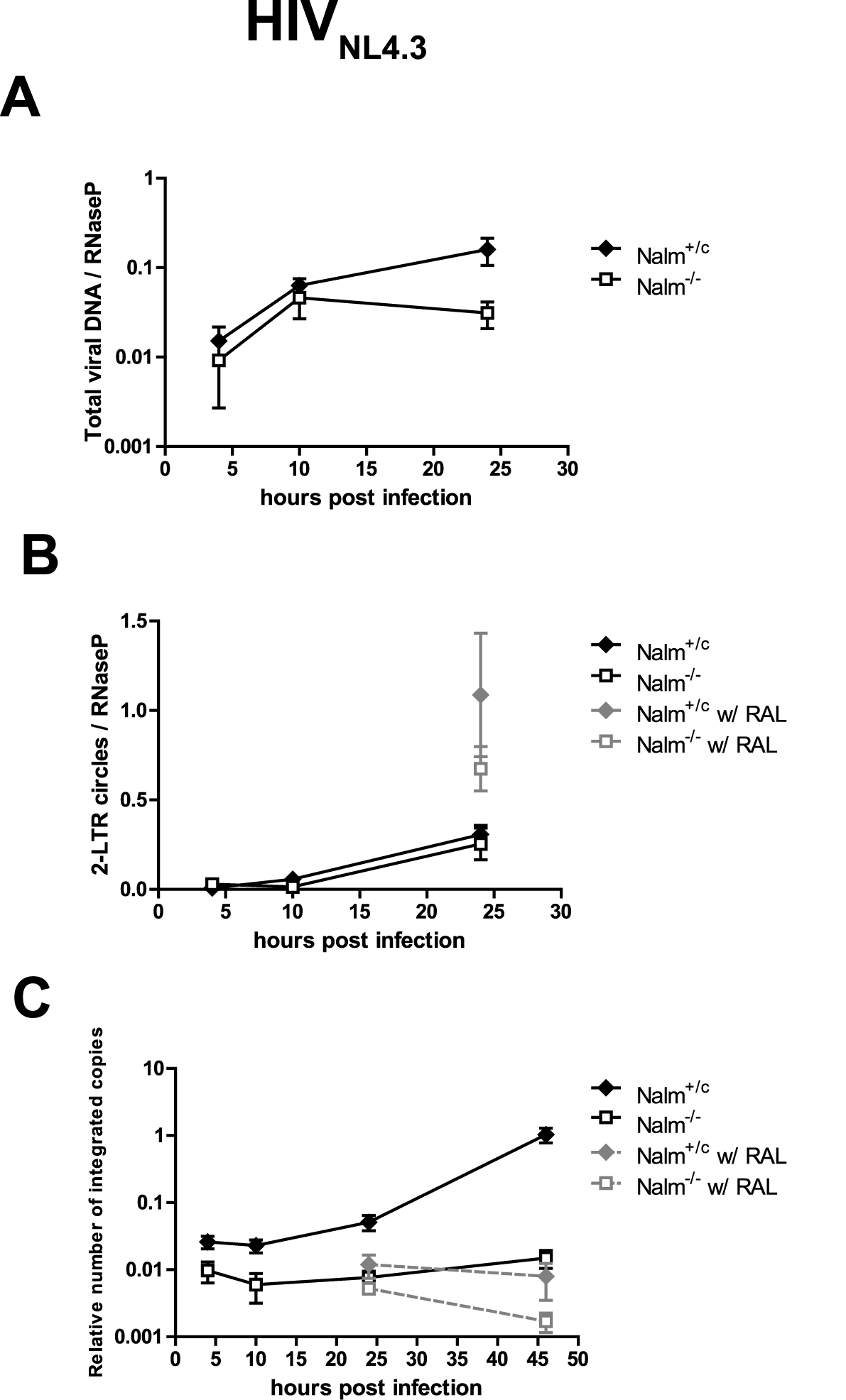

Supplement: Figure S3 — Analysis of HIV-1 DNA species in Nalm+/c and Nalm−/− cell lines at different time points after infection with HIVNL4.3. (A) Relative number of total viral DNA, (B) 2-LTR circles and (C) integrated proviral copies (Alu-qPCR). Experiments performed in duplicate and analyzed with qPCR in triplicate. Copy number ratio normalized to RNaseP of a representative experiment is shown with standard deviations. RAL was used at 50 times IC50 concentration. (TIF) [file ppat.1002558.s003.tif]

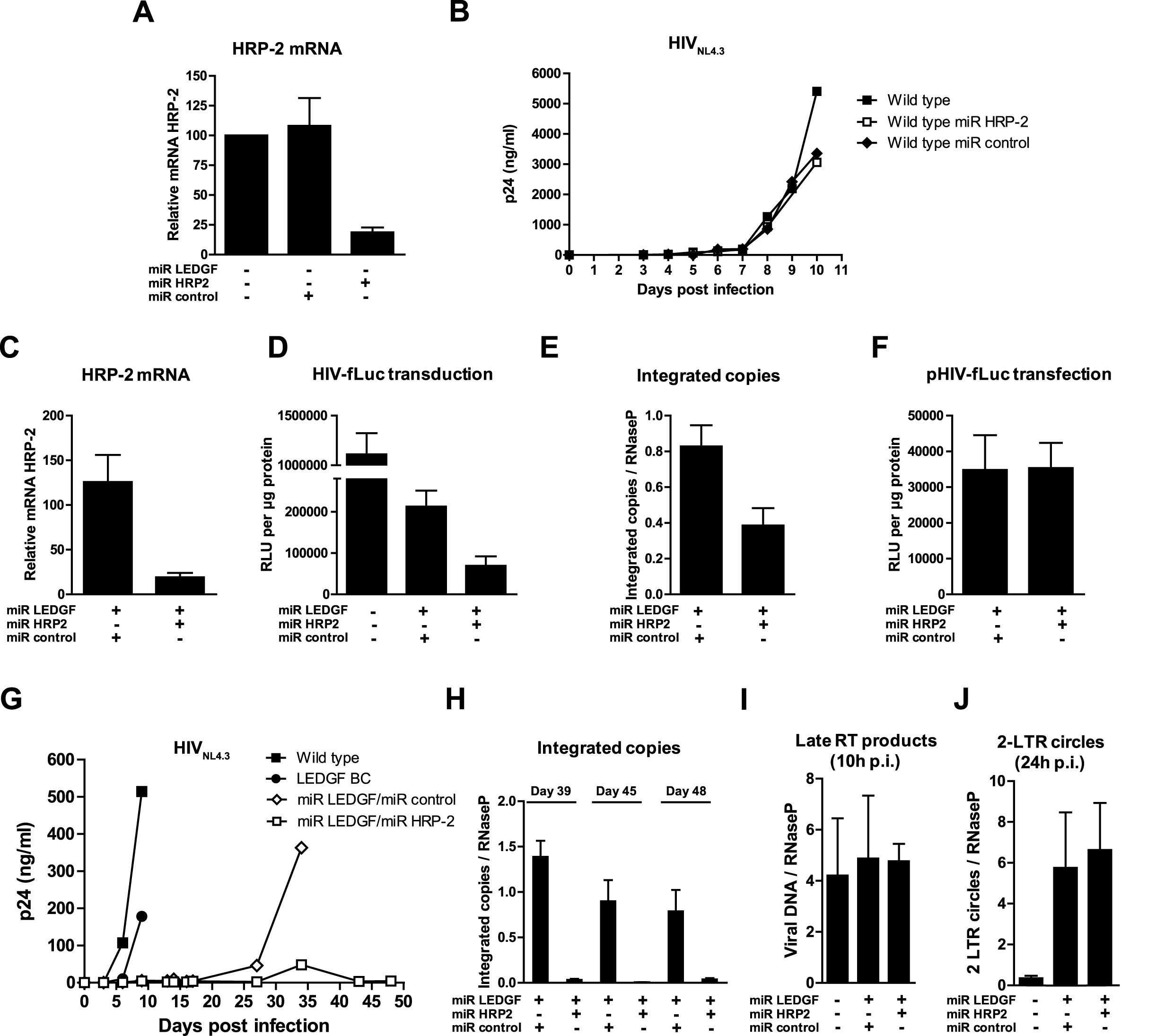

Supplement: Figure S4 — Additional HRP-2 KD blocks residual HIV-1 replication in LEDGF/p75 KD cells. (A–B) Multiple round HIV-1 replication in HeLaP4 HRP-2 KD cells (wild-type miR HRP-2) and control cell lines (wild-type and wild-type miR control). (A) HRP-2 mRNA levels were determined by qPCR, normalized to RNaseP expression levels and expressed as percentage from wild-type. (B) Cells were challenged with HIVNL4.3 and supernatant was harvested for p24 ELISA. Experiments were performed in duplicate; a representative experiment is shown. (C–J) Additional HRP-2 KD (miR LEDGF/miR HRP-2) and control (miR LEDGF/miR control) cell lines were generated from stable LEDGF/p75 KD HeLaP4 cell lines (miR LEDGF). Constructs used to generate the cells are listed below the graph. (C) HRP-2 mRNA expression levels were determined with qPCR, normalized to RNaseP expression levels and expressed as percentage from wild-type. (D) Different cell lines were transduced with HIV-fLuc and luciferase expression was quantified (RLU per µg protein). In (E) the number of integrated copies was determined. Following transduction with HIV-fLuc, cells were grown for an additional 10 days to eliminate non-integrated viral DNA. (F) Different HeLaP4 cell lines were transfected with pHIV-fLuc and luciferase expression was quantified. In panel (G) we challenged the different cell lines with the laboratory strain HIVNL4.3. The experiment was continued for 48 days. Supernatant was harvested for p24 ELISA. Experiments were performed in duplicate; a representative experiment is shown. (H) Following multiple round HIV-1 infection as presented in (G), we determined the number of proviral copies by qPCR in miR LEDGF/miR control and miR LEDGF/miR HRP-2 cells on day 39, 45 and 48, when cells were grown for an additional 10 days in the presence of antiretroviral therapy to eliminate non-integrated viral DNA. In (I) we determined late reverse transcripts (Late RT products) at 10 hrs post infection (p.i.) with HIVNL4.3 using qPCR, normalize [file ppat.1002558.s004.tif]
