# Supplementary material for: LEDGF/p75-Independent HIV-1 Replication Demonstrates a Role for HRP-2 and Remains Sensitive to Inhibition by LEDGINs
Source: PLoS Pathog. 2012 Mar 1;8(3):e1002558. doi: 10.1371/journal.ppat.1002558 (PMC3291655; doi:10.1371/journal.ppat.1002558)
Supplement: Table S1 — IC50 values for LEDGINs (LEDGIN 7) and INSTIs (RAL). IC50 values for LEDGIN 7 and Raltegravir were calculated based on data shown in figure 6. Data were fitted to a sigmoidal dose-response (variable slope) curve, from which IC50 values were calculated. Mean and 95% confidence interval are shown. (DOC) [file ppat.1002558.s008.doc]

| **Table S1. IC_50_ values for LEDGINs (LEDGIN 7) and INSTIs (RAL)** | | | |
| --- | --- | --- | --- |
| **Inhibitor** | **Cell line** | **IC_50_ (µM)** | **95% CI** |
|  |  |  |  |
| **LEDGIN 7** | Nalm^+/c^ | 0.46 | 0.27 to 0.78 |
|  | Nalm^-/-^ | 0.18 | 0.10 to 0.31 |
|  |  |  |  |
| **Raltegravir** | Nalm^+/c^ | 0.0027 | 0.0019 to 0.0029 |
|  | Nalm^-/-^ | 0.0020 | 0.0016 to 0.0027 |
|  |  |  |  |
| *Abbreviations:* IC_50_, half maximal inhibitory concentration; INSTI, Integrase strand transfer inhibitor; RAL, Raltegravir; CI, confidence interval. | | | |
