# Supplementary material for: LEDGF/p75-Independent HIV-1 Replication Demonstrates a Role for HRP-2 and Remains Sensitive to Inhibition by LEDGINs
Source: PLoS Pathog. 2012 Mar 1;8(3):e1002558. doi: 10.1371/journal.ppat.1002558 (PMC3291655; doi:10.1371/journal.ppat.1002558)
Supplement: Table S2 — Overview of primers and probes used. Primers and probes used throughout the manuscript are shown. (DOC) [file ppat.1002558.s009.doc]

| **Table S2. Overview of primers and probes used** | | | |  |
| --- | --- | --- | --- | --- |
| **Name** | **Sequence** |  | |  |
| LEDGF/p75 attB1 | 5′-(attB1) GACTGCAAAGGAACTGACTGTAGC-3' | |  | |
| LEDGF/p75 attB2 | 5′-(attB2) AGTTTCACTGTGTATACGCCACAG-3' | |  | |
| LEDGF/p75 attB3 | 5′-(attB3) GAATTCAGTGGCAAAGTCACTGTC-3' | |  | |
| LEDGF/p75 attB4 | 5`-(attB4) TGAGAGGATGAAATTGACTGAGGC-3` | |  | |
| A | 5’-ACTTCTCAAGTGTTCTCTATATTCCAGG-3’ | |  | |
| B | 5’-TCGAAGAGGTTCACTAGTACTGGCCATTGC-3’ | |  | |
| C | 5’-ATGTCATGATAATAATGGTTT CTTAGACGTGCG-3’ | |  | |
| D | 5'-ACTGCACCATTCTAAGAGCT-3' | |  | |
| E | 5'-CTTTGTATACTTTTTCTGTG-3' | |  | |
| gFB | 5’-GGCCTGTTTGTCCAGTGACC-3’ | |  | |
| gRB | 5’-GAGTCATCTGCCTCATGAGC-3’ | |  | |
| gFA | 5’-TTTATGCTACGGAACTGCACC-3’ | |  | |
| gRA | 5’-CACTGCTCTAGTCCTTCAATAGGC-3’ | |  | |
| RNA-A | 5'-CCCGGAAACATGACTCGCG-3' | |  | |
| RNA-B | 5'-TCATTAAAACCTTTTCTTTTATTTGG-3' | |  | |
| RNA-C | 5’-ACTTCTCAAGTGTTCTCTATATTCCAGG-3’ | |  | |
| RNA-D | 5'-CTACTGTAGATTACATGTTGTTTG-3' | |  | |
| RNA-E | 5′-GAACTTGCTTCACTTCAGGTC-3′ | |  | |
| RNA-F | 5′-TCGCCGTATTTTTTTCAGTGT-3′ | |  | |
| LEDGF probe4 | 5′-FAM-TGCAACAAGCTCAGAAACACACAGAGATGA-TAMRA-3′ | |  | |
| d243 | 5’-AAGCAAGAAAAGAGCCG-3’ | |  | |
| d244 | 5’-TGTTGGATCCAGAAAAGAGCCGGAT-3’ | |  | |
| LEDGF-R-exon15 | 5’-GGCTTCATGGTTGTCTTTGC-3’ | |  | |
| LPROBE-Fwd | 5'-GTACCTTTCCAATAACCCCATCTGTCTAG-3' | |  | |
| LPROBE-Rev | 5'-CACTTCGTTTAAATTCAAAAGTAATTCACA-3' | |  | |
| Zeo-Fwd | 5’-CAAGCTAGCATCGATATGGCCAAGTTGACCAG-3’ | |  | |
| Zeo-Rev | 5’-GGTCGTACGTTCAGTCCTGCTCGTCGGCC-3’ | |  | |
| Hygro-Fwd | 5'-AAAAAAATCGATGAAAAAGCCTGAACTCACCGCG-3' | |  | |
| Hygro-Rev | 5'-ACTAGTGTCGACCTATTCCTTTGCCCTCGGACG-3' | |  | |
| HRP2-Fwd | 5’-TTCGGATCCGAGCGGACCCGGAAGCGG-3’ | |  | |
| HRP2-Rev | 5’-TTCAAGCTTTCAGCTCTCCTCGTCCAGGGCCTCC-3’ | |  | |
| CD4-Fwd | 5'-ATAGGATCCATGAACCGGGGAGTCCC-3' | |  | |
| CD4-Rev | 5'-ATATCTAGATTCAAATGGGGCTACATGTCTTC-3' | |  | |
| HRP2 s4 | 5′-AGGATGGAGAGCGACTCAGA-3′ | |  | |
| HRP2 as4 | 5′-CGAGTTCTCCTCTTCGGAT-3’ | |  | |
| HRP2 probe | 5’-FAM-GCGCTAAAGATGTCGGTCTC-TAMRA-3’ | |  | |
| AluSINIIfwd | 5'-GTGGAGGTTTGACAGCCGCCTAGC-3' | |  | |
| qAluRout_SB704 | 5'-TGCTGGGATTACAGGCGTGAG-3' | |  | |
| Q-Alu-F-in | 5'-AGCTTGCCTTGAGTGCTTCAA-3' | |  | |
| Q-Alu-R-in | 5'-TGACTAAAAGGGTCTGAGGGATCT-3' | |  | |
| nested Alu-probe | 5’-FAM-TTACCAGAGTCACACAACAGACGGGCA-TAMRA-3’ | |  | |
|  |  | |  | |
|  | **shRNA targeting sequence on mRNA** | |  | |
| miR HRP2 | 5'-GAAGCTGCACAGTGAGATCAAG-3' | |  | |
| miR scrambled | 5'-TGATGGCAACAATATCCACAAG-3' | |  | |
| miR DsRed | 5'-AAATGCAGAAGAAGACCATGG-3' | |  | |
