# Supplementary material for: LEDGF/p75-Independent HIV-1 Replication Demonstrates a Role for HRP-2 and Remains Sensitive to Inhibition by LEDGINs
Source: PLoS Pathog. 2012 Mar 1;8(3):e1002558. doi: 10.1371/journal.ppat.1002558 (PMC3291655; doi:10.1371/journal.ppat.1002558)
Supplement: Table S3 — Effect of LEDGF/p75 KD or KO on the frequency of viral integration in genomic features. Comparison of the HIV integration site distribution pattern elaborated in current and previous publications [2], [11], [17]. Frequency of viral integration in genomic features (integration in RefSeq genes, ±2 kb or ±4 kb around CpG islands) is shown. (DOC) [file ppat.1002558.s010.doc]

Table S3. Effect of LEDGF/p75 KD or KO on the frequency of viral integration in genomic features

| **Species** | **Virus or**  **vector** | **Cell line** | **Specifications** | **Alias** | **Total**  **Sites** | **In RefSeq genes (%)** | ***P* value^1^** | ∆^2^ | **±2kb of CpG islands(%)** | ***P* value^3^** | **±4kb of CpG islands(%)** | ***P***  **Value^4^** | **MRC sites** | **MRC, In RefSeq genes (%)^5^** | **MRC, ±2kb CpG (%)^5^** | **MRC, ±4kb CpG (%)^5^** | **Source** |
| --- | --- | --- | --- | --- | --- | --- | --- | --- | --- | --- | --- | --- | --- | --- | --- | --- | --- |
| Human | HIV | HOS | Control | U6 | 106 | 73.58 |  |  | 1.89 |  | 5.66 |  | 318 | 42.77*** | 6.29 | 8.18 | [1] |
|  |  |  | LEDGF/p75 KD | siL | 189 | 71.96 | 0.8 | 1.62 | 1.59 | 1.0 | 7.41 | 0.6 | 567 | 42.15*** | 3.35 | 6.00 | [1] |
|  |  | HEK293T | Control | 293T-siScram | 449 | 70.38 |  |  | 1.11 |  | 4.90 |  | 4490 | 41.63*** | 4.21*** | 6.95 | [1] |
|  |  |  | LEDGF/p75 KD | 293T-siLL | 592 | 61.99 | 0.06 | 8.39 | 6.25 | **<0.0001** | 13.34 | **<0.0001** | 5920 | 40.42*** | 3.61** | 6.30*** | [1] |
|  |  | Jurkat | Control | Jurkat-siJK2BC | 682 | 79.62 |  | 5.95 | 2.64 |  | 8.50 |  | 6819 | 40.42*** | 4.21 | 6.94 | [1] |
|  |  |  | LEDGF/p75 KD | Jurkat-siJK2 | 695 | 73.67 | **0.01** |  | 3.88 | 0.2 | 11.37 | 0.09 | 6950 | 40.49*** | 4.19 | 6.63*** | [1] |
|  |  | Nalm-6 | Control | Nalm^+/+^, Nalm^+/c^ | 1736 | 77.50 |  |  | 4.49 |  | 11.81 |  | 5208 | 39.80*** | 3.00** | 5.39*** | This work |
|  |  |  | LEDGF/p75 KO | Nalm^-/-^ cl 1, 2 | 799 | 51.80 | **<0.0001** | 25.7 | 9.89 | **<0.0001** | 15.14 | **0.02** | 2397 | 39.13*** | 3.38*** | 5.55*** | This work |
| Mouse | HIV | MEF | Control | E1^f/+^ | 341 | 72.14 |  |  | 2.64 |  | 7.04 |  | 1023 | 38.51*** | 3.23 | 6.16 | [2] |
|  |  |  | LEDGF KO | E2^-/-^ | 434 | 50.23 | **<0.0001** | 21.91 | 6.91 | **0.008** | 12.9 | **0.009** | 1302 | 40.25*** | 3.46** | 6.22** | [2] |
|  |  | MEF | Control | MEF +/+ | 1105 | 64.25 |  |  | 1.45 |  | 4.89 |  | 11050 | 36.17*** | 2.14 | 4.06 | [3] |
|  |  |  | LEDGF KO | MEF -/- | 495 | 50.10 | **<0.0001** | 14.15 | 7.27 | **<0.0001** | 11.52 | **<0.0001** | 4950 | 36.77*** | 2.12*** | 3.90*** | [3] |

^1^ *P* value obtained with a two-tailed Fisher’s exact test for the difference for integration in RefSeq genes obtained using HIV in control compared to LEDGF/p75 KD or KO (in MEF denoted as LEDGF KO) cell lines.

^2^ Delta (∆) represents the difference of integration in RefSeq genes in control compared to KD or KO conditions

^3^Comparable to (^1^), except integration **±**2kb of CpG islands is used as a genomic feature.

^4^ Comparable to (^1^), except integration **±**4kb of CpG islands is used as a genomic feature.

^5^ Experimentally derived integration sites were compared to MRC using Fisher’s exact test. Statistical difference was denoted as: * *P*<0.05, ** *P*<0.01, *** *P*<0.001.

**REFERENCES**

1. Ciuffi A, Llano M, Poeschla E, Hoffmann C, Leipzig J, et 684 al. (2005) A role for LEDGF/p75 in targeting HIV DNA integration. Nat Med 11: 1287-1289.

2. Shun MC, Raghavendra NK, Vandegraaff N, Daigle JE, 708 Hughes S, et al. (2007) LEDGF/p75 functions downstream from preintegration complex formation to effect gene specific HIV-1 integration. Genes Dev 21: 1767-1778.

1. Marshall HM, Ronen K, Berry C, Llano M, Sutherland H, et al. (2007) Role of PSIP1/LEDGF/p75 in lentiviral infectivity and integration targeting. PLoS One 2: e1340.
